# Scaffold-Scaffold Interaction Facilitates Cell Polarity Development in Caulobacter crescentus

**DOI:** 10.1128/mbio.03218-22

**Published:** 2023-03-27

**Authors:** Ning Lu, Samuel W. Duvall, Guohong Zhao, Kimberley A. Kowallis, Chao Zhang, Wei Tan, Jingxian Sun, Haley N. Petitjean, Dylan T. Tomares, Guo-Ping Zhao, W. Seth Childers, Wei Zhao

**Affiliations:** a CAS Key Laboratory of Quantitative Engineering Biology, Shenzhen Institute of Synthetic Biology, Shenzhen Institute of Advanced Technology, Chinese Academy of Sciences, Shenzhen, China; b Department of Chemistry, University of Pittsburgh, Pittsburgh, Pennsylvania, USA; c Department of Pharmacy, School of Life Sciences, Henan University, Kaifeng, China; d CAS Key Laboratory of Synthetic Biology, CAS Center for Excellence in Molecular Plant Sciences, Chinese Academy of Sciences, Shanghai, China; e State Key Lab of Genetic Engineering and Institutes of Biomedical Sciences, Department of Microbiology and Microbial Engineering, School of Life Sciences, Fudan University, Shanghai, China; University of Wyoming; Fred Hutchinson Cancer Center

**Keywords:** *Caulobacter crescentus*, asymmetric cell division, cell polarity, scaffold proteins, PodJ, PopZ, chromosome segregation

## Abstract

Cell polarity development is the prerequisite for cell differentiation and generating biodiversity. In the model bacterium Caulobacter crescentus, the polarization of the scaffold protein PopZ during the predivisional cell stage plays a central role in asymmetric cell division. However, our understanding of the spatiotemporal regulation of PopZ localization remains incomplete. In the current study, a direct interaction between PopZ and the new pole scaffold PodJ is revealed, which plays a primary role in triggering the new pole accumulation of PopZ. The coiled-coil 4-6 domain in PodJ is responsible for interacting with PopZ *in vitro* and promoting PopZ transition from monopolar to bipolar *in vivo*. Elimination of the PodJ-PopZ interaction impairs the PopZ-mediated chromosome segregation by affecting both the positioning and partitioning of the ParB-*parS* centromere. Further analyses of PodJ and PopZ from other bacterial species indicate this scaffold-scaffold interaction may represent a widespread strategy for spatiotemporal regulation of cell polarity in bacteria.

## INTRODUCTION

**FIG 1:**
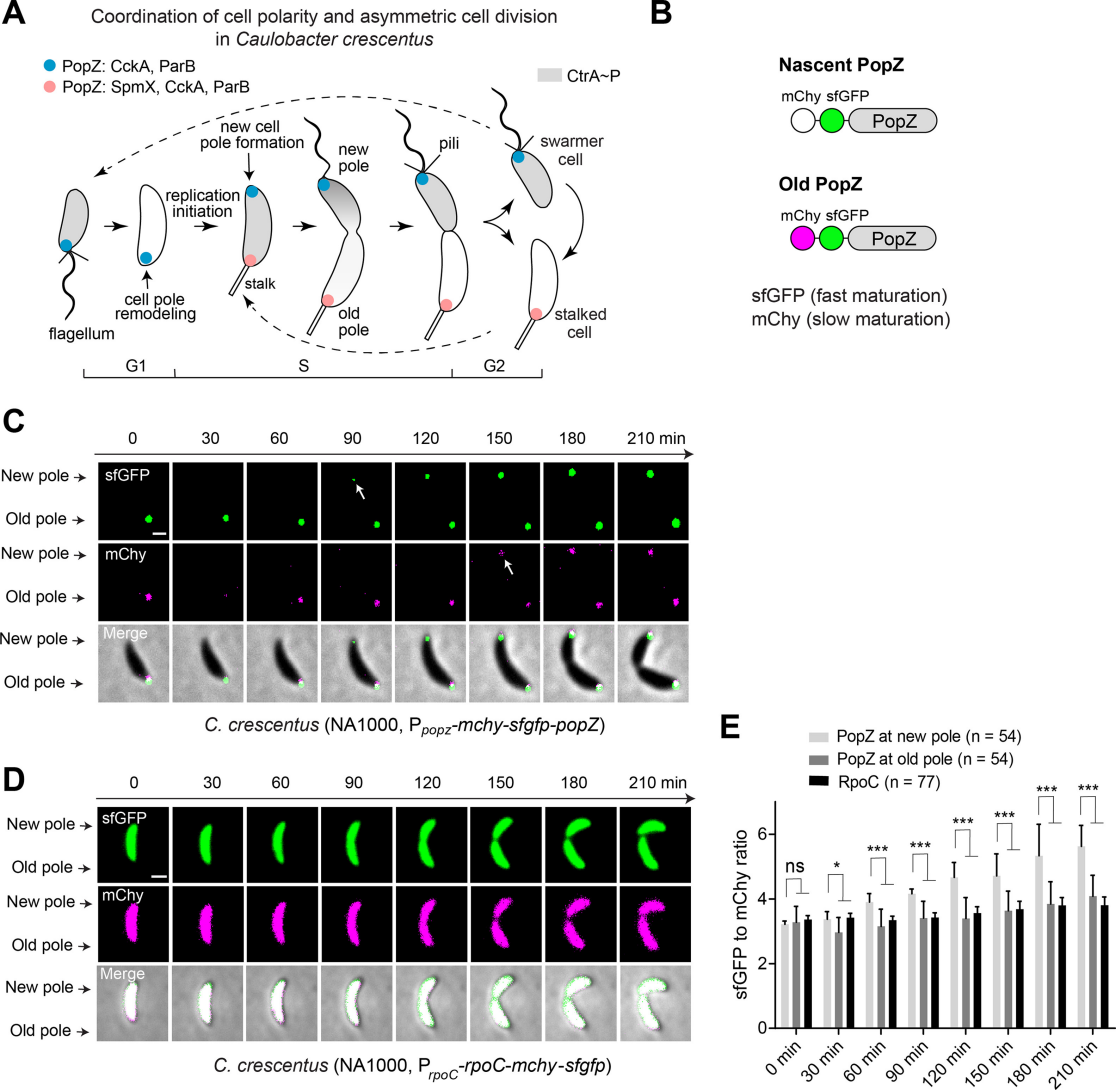
PopZ polarizes at the new cell pole through *de novo* protein synthesis. (A) Schematics of cell polarity development in C. crescentus. PopZ organizes distinct signaling proteins at opposite cell poles during the cell cycle. As the new pole assembles, PopZ protein accumulates gradually upon initiation of replication, which is associated with the formation of the new pole signaling hub. The polarization of PopZ results in asymmetric cell division that generates daughter cells with distinct cell fates. (B) Construction of the tandem fluorescent-tagged PopZ. PopZ fused to two tandem fluorescent proteins: one matures rapidly (sfGFP, *t*_50_ = 19 min at 32°C), and one matures slowly (mCherry, *t*_50_ = 45 min at 32°C). The presence of high sfGFP fluorescence and weak mCherry fluorescence represents newly synthesized protein (32). (C) PopZ accumulates at the new pole through *de novo* synthesis. The tandem mCherry-sfGFP-PopZ is expressed under the native *popZ* promoter in the C. crescentus NA1000 genome. Time-lapse microscopy was used to detect the fluorescent signal of PopZ in cells after synchronization at 28°C on the PYE agarose pad containing kanamycin. Newly synthesized PopZ appears green, followed by a transition to “white” as the “magenta” mCherry matures, which is indicated with white arrows. One representative cell is shown here (see more cells in Fig. S1B). (D) The constitutively expressed RpoC (32) displays a constant sfGFP-to-mCherry ratio during the cell cycle. (E) Quantification of the average sfGFP-to-mCherry fluorescence ratios for PopZ during the cell cycle. The fluorescent signals of mCherry-sfGFP-PopZ at both cell poles were measured. The background-subtracted sfGFP and mCherry intensities were counted using Fiji/ImageJ (49). The sfGFP-to-mCherry ratio was calculated for each cell and the average sfGFP-to-mCherry ratios were measured for each time point. Each experiment was performed in four biological replicates. At least 50 cells (*n*) in total were calculated in each sample. ***, *P < *0.01; *, *P < *0.05; ns, *P ≥ *0.05 by Welch’s unpaired *t* test. All scale bars = 1 μm.

Cell polarity refers to the intrinsic asymmetry of cells, either at the level of morphology, structure, or organization of cellular components, and is a fundamental means for speciation and biodiversity (1, 2). Studies have demonstrated that cell polarization occurs both in eukaryotes and prokaryotes (3–5). In bacteria, Caulobacter crescentus is a well-established model for studying cell polarity development and asymmetric cell division. Two morphologically and functionally distinct cells are generated at each cell division: a motile swarmer cell and a sessile stalked cell (1, 6). The swarmer cell differentiates into the stalked cell and reenters the cell cycle by shedding the flagellum and initiating stalk biogenesis and chromosome replication (Fig. 1A). The two daughter cells utilize bimodal survival strategies, showing differences in mobility (7) and buoyancy (8) and distinct responses to heavy metal stress (9). Previous studies have indicated that C. crescentus does not have protein orthologs responsible for driving eukaryotic stem cell division but rather employs a set of divergent scaffold proteins to achieve cell polarization (1, 10, 11).

10.1128/mbio.03218-22.1 FIG S1 Pole-specific FRAP analysis suggests that the newly synthesized PopZ could be accumulated at both cell poles. (A) Expression of mCherry-sfGFP-PopZ shows a bipolar localization pattern as that of the untagged PopZ (19) in predivisional C. crescentus cells. In the predivisional cell stage after synchronization, the subcellular localization of mCherry-sfGFP-PopZ was monitored in LN001 (NA1000, P*_popZ_*-*mcherry*-*sfgfp*-*popZ*) strain. The fluorescence tagged *popZ* gene was integrated in the chromosome of C. crescentus under the control of the native promoter. (B) PopZ accumulates at the new pole through *de novo* synthesis. Two more cells are shown here as in Fig. 1C. (C) FRAP analysis reveals that the fluorescence intensities of mCherry-sfGFP-PopZ condensates were partially recovered after photobleaching in C. crescentus. The expression of mCherry-sfGFP-PopZ was driven by the endogenous *popZ* promoter in the C. crescentus chromosome. The cell pole regions with all the PopZ accumulated were selected for photobleaching (indicated by the white circles). One representative bleached cell is shown. White arrows indicate the fluorescence recovery at the new cell pole or old cell pole. (D) Quantification of the FRAP analyses in panel A. The recovery curve was generated by averaging the signals of 7 cells. A total of 27% and 15% recovery within 3 minutes were shown at the new cell pole and the old cell pole, respectively. The fluorescence intensity of prebleached foci was normalized as 100%. Taking the average fluorescent signal of three PopZ foci without bleaching as a reference, each signal intensity of the experimental group was first normalized with the signal intensity of the reference group at each time point. ***, *P < *0.001 determined by two-way ANOVA. All scale bars = 1 μm. Download FIG S1, PDF file, 0.3 MB. Copyright © 2023 Lu et al. 2023 Lu et al. https://creativecommons.org/licenses/by/4.0/ This content is distributed under the terms of the Creative Commons Attribution 4.0 International license.

Scaffold proteins have shown the ability to direct and rewire cellular information flow by coordinating the locations of diverse signaling proteins in cells (12, 13). Through linking to macromolecular assemblies, scaffold proteins are involved in biomolecular condensate formation, cytoskeletal dynamics, cell polarity, and cell division (12, 14). In C. crescentus, a set of spatiotemporally distributed scaffold-signaling complexes are essential for establishing and maintaining cell polarity (14–17). Among these, scaffold PopZ plays a central role in organizing the polar signaling hubs. These include the old pole scaffold protein SpmX and histidine kinase DivJ (14), the bipolar histidine kinase CckA, and the modulator DivL (15). PopZ also serves as an attachment site for the ParB-*parS* centromere during chromosome segregation (15, 18, 19). The timing of the new pole accumulation of PopZ is strictly correlated with ParB-*parS* centromere partitioning (17, 20). Single-molecule tracking experiments (21) and fluorescence loss in photobleaching assays (15) have shown that PopZ dynamically recruits distinct protein clients at each cell pole in predivisional cells. The polarization of PopZ complexes by redistribution of PopZ from monopolar to bipolar localization pattern is pivotal for polar signaling differentiation and cell fate determination (11, 15) (Fig. 1A). Nevertheless, the spatiotemporal regulation of scaffold PopZ during the cell cycle remains largely unknown.

Previous studies have revealed that the zinc finger protein ZitP (22), the new pole marker protein TipN (17), and the chromosome segregation ATPase ParA (17) are associated with PopZ new pole accumulation. Both ZitP and ParA were shown to interact with PopZ directly (17, 23). Deletion of *zitP* mildly reduced the bipolar localization of PopZ (22), while deletion of *tipN* delayed the arrival of PopZ at the new cell pole (17). Nevertheless, most of these reports were based on mutations or truncations of PopZ rather than manipulation of the possible upstream regulators. Other elements that might be needed for the robust bipolar localization of PopZ have been indicated (17, 22). One potential candidate is the scaffold protein PodJ, which has been shown to selectively localize at the new cell pole (24, 25). PodJ is a transmembrane protein that contains a cytoplasmic N terminus composed of a coiled-coil-rich region and an intrinsically disordered region, followed by a C terminus that involves pili biogenesis (26, 27). Previous reports have suggested that PodJ recruits phosphatase PleC and adaptor protein PopA to the new cell pole (16, 25, 27–30). Deletion of *podJ* results in reduced PilA protein levels (16, 25, 26), downregulation of the CtrA signaling pathway (16, 26), and moderate loss of new pole localization of PopZ client proteins (16). In the current study, by applying a combination of genetics, quantitative cell biology, and biochemical assays, we demonstrate that PodJ functions as a primary regulator in triggering PopZ new pole accumulation compared with other factors. The physical interaction between PopZ and PodJ ensures the robust accumulation of PopZ at the new cell pole. Deletion of *podJ* impairs the ability of PopZ to tether the chromosome segregation protein ParB at the new cell pole. We propose that the PodJ-PopZ interaction is implicated in the stringent inheritance of the polarity axis and spatiotemporal coordination of cell polarity development.

## RESULTS

### PopZ accumulates at the new cell pole by *de novo* synthesis.

Cell polarization of C. crescentus is accompanied by the transition of PopZ from old polar accumulation to bipolar accumulation (11, 15) (Fig. 1A). We were curious whether the redistribution of this scaffold resulted from subcellular migration or protein new synthesis. To answer this question, a fluorescent timer cassette was constructed by in-frame and simultaneous integration of the slow-maturing mCherry and the fast-maturing superfolder green fluorescent protein (sfGFP) (31) to the chromosome after the endogenous *popZ* promoter (P*_popz_*-*mchy-sfgfp-popZ*) (Fig. 1B). Expressing the fusion protein of mCherry-sfGFP-PopZ did not change the bipolar localization pattern of PopZ in predivisional C. crescentus cells (Fig. S1A in supplemental material). As described previously (31, 32), the high sfGFP and weak mCherry fluorescence signal represents a newly translated protein, while the high sfGFP and high mCherry fluorescence signal represents a formerly translated protein. Time-course imaging on synchronized C. crescentus cells expressing mCherry-sfGFP-PopZ revealed that the new pole PopZ exhibited a significant sfGFP signal about 60 min ahead of the mCherry signal. Moreover, an increasing sfGFP-to-mCherry ratio was observed for the new pole PopZ during the predivisional stage, compared to that of PopZ at the old cell pole (Fig. 1C and E and Fig. S1B). As a negative control, the constitutively expressed β′ subunit of RNA polymerase (RpoC) displayed a steady-state level of sfGFP-to-mCherry ratio when fused to the same fluorescence timer cassette (Fig. 1D and E and Table S1). These observations indicate that the new cell pole is populated with newly synthesized mCherry-sfGFP-PopZ while the older mCherry-sfGFP-PopZ is retained at the old cell pole.

10.1128/mbio.03218-22.8 TABLE S1 The raw data of fluorescence intensity of PopZ at the new cell pole and old cell pole. Download Table S1, XLSX file, 0.02 MB. Copyright © 2023 Lu et al. 2023 Lu et al. https://creativecommons.org/licenses/by/4.0/ This content is distributed under the terms of the Creative Commons Attribution 4.0 International license.

To further test if the newly synthesized PopZ could accumulate at the old cell pole as well, a pole-specific photobleaching experiment was performed. Fluorescence recovery after photobleaching (FRAP) analysis demonstrated that the fluorescence intensity of the bleached mCherry-sfGFP-PopZ could be partially recovered at both cell poles (Fig. S1C and D). However, a relatively slower recovery rate was observed at the old cell pole (15% versus 27% recovery within 3 min at the opposite poles), which implies an inequal PopZ accumulation at the opposite poles during the predivisional stage. Taken together, these observations indicate that the new pole accumulation of PopZ resulted from *de novo* protein synthesis rather than migration from the old cell pole.

### PodJ is the primary regulator that triggers PopZ accumulation at the new cell pole.

**FIG 2:**
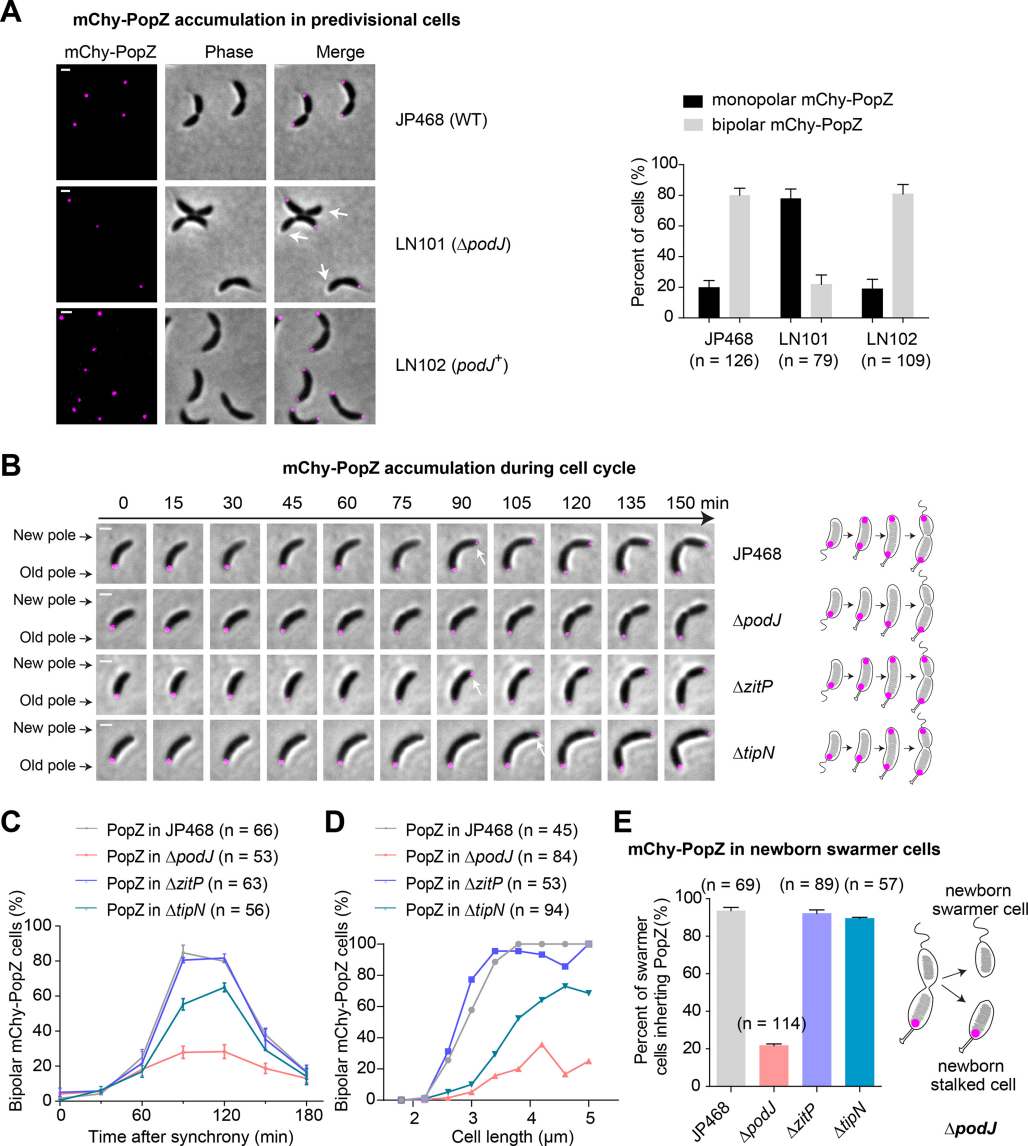
PodJ promotes the new pole accumulation of PopZ in C. crescentus. (A) Deletion of *podJ* results in decreased bipolar accumulation of mCherry-PopZ. In the predivisional cell stage after synchronization, the subcellular localization of mCherry-PopZ was monitored in JP468 (NA1000, P*_popZ_-mcherry-popZ*, and P*_parB_-cfp-parB*) (23), LN101 (NA1000 Δ*podJ*, P*_popZ_-mcherry-popZ*, and P*_parB_-cfp-parB*), and LN102 (NA1000 Δ*podJ*, P*_podJ_*-*podJ*, P*_popZ_-mcherry-popZ*, and P*_parB_-cfp-parB*) strains. A sole copy of *popZ* in these strains was expressed under the control of the native promoter. Quantification of the cells with different mCherry-PopZ localization patterns is shown on the right. The observation of the stalk at the cell pole is considered the old cell pole. White arrows indicate the new cell poles. (B) Time-lapse microscopy indicates that Δ*podJ* causes a significant delay in mCherry-PopZ accumulation at the new pole. Cells of JP468, Δ*podJ*, Δ*zitP*, and Δ*tipN* were synchronized and transferred onto an M2G agarose pad before observation. The mCherry-PopZ signal was visualized at 15 min intervals at 30°C. The white arrow indicates the time points when the signal of mCherry-PopZ was first detected. The localization patterns of mCherry-PopZ over time are shown as schematics on the right. (C) Quantitative analyses suggest that PodJ plays a primary role in triggering bipolar PopZ. Cells were synchronized and transferred to a liquid M2G medium for continuous culture before observation. At least 50 cells (*n*) were calculated in each sample. (D) Quantitative analyses of bipolar mCherry-PopZ cells using cell length as the marker of cell cycle progression. All cells from all time points were sorted by cell length, and the fraction of cells with bipolar mCherry-PopZ is shown for each bin of 0.4 μm. (E) Disruption of the PodJ-PopZ interaction results in a failure to inherit PopZ in ~80% of swarmer cells. The schematic of mCherry-PopZ localization in Δ*podJ* is shown on the right. Each experiment was performed in three biological replicates. Measurements are shown as mean ± standard deviations. All scale bars = 1 μm.

PopZ shows bipolar localization in predivisional C. crescentus (19), while heterologous expression of PopZ results in monopolar accumulation in Escherichia coli (15). A key question is what triggers the accumulation of newly synthesized PopZ at the new pole in C. crescentus. To answer this, the identified new pole scaffold PodJ (24, 25) was used to test the potential interaction with PopZ. PodJ accumulates at the new pole slightly earlier than PopZ during the cell cycle (32). In-frame deletion of *podJ* in C. crescentus resulted in ~80% of cells did not show accumulation of mCherry-PopZ at the new cell poles at the predivisional cell stage (~60% decrease of the cells compared to the wild-type cells) (Fig. 2A). In contrast, the complementation of *podJ* recovered the new pole PopZ accumulation in 85% of cells, which was comparable to wild-type C. crescentus, indicating that PodJ plays a role in promoting the new pole accumulation of PopZ. Supporting this result, time-lapse microscopy experiments starting with a synchronized population of Δ*podJ* swarmer cells revealed significantly delayed signals for mCherry-PopZ at the new poles (Fig. 2B to D). A subset of Δ*podJ* cells (~75% of the cells) lacked any observable PopZ foci even after cell division (Fig. 2E). Nevertheless, the PopZ localization to the flagellated pole is severely delayed but may not be fully inhibited, and in the majority of cases PopZ still ends up accumulating at the flagellated pole by the swarmer cell stage (data not shown).

Previous studies have suggested that ZitP (22) and TipN (17, 33) are directly associated with PopZ accumulation at the new pole. ZitP is a zinc finger protein that is required for pilus biogenesis and cell motility (34). TipN regulates the dynamics of chromosome segregation by interacting with ParA at the new cell pole (35, 36). In the current study, the effects of ZitP and TipN upon PopZ accumulation were tested in parallel with PodJ (Fig. 2B). Quantification of the time-lapse microscopy results indicates that deletion of *zitP* had no significant impact on the new pole accumulation of PopZ, and deletion of *tipN* resulted in a minor delay of the new pole accumulation of PopZ, compared to that of Δ*podJ* (Fig. 2C). To exclude possible effects from cell growth defects by the gene deletions, the new pole accumulation of PopZ was evaluated using cell length instead of time as the marker of cell cycle progression in these genetically null mutant strains. A similar result was observed that PodJ had the most significant impact on the bipolarization of PopZ in C. crescentus (Fig. 2D). The delayed new pole accumulation of PopZ in Δ*podJ* was not caused by the reduced expression of *popZ* (Fig. S2A to C). Western blotting analyses suggest that the expression level of mCherry-PopZ did not change noticeably in the genetically null strains, including Δ*podJ* (Fig. S2C). Collectively, these results indicate that PodJ plays a primary role in triggering PopZ accumulation at the new pole, compared with other protein regulators.

10.1128/mbio.03218-22.2 FIG S2 The PodJ effect upon PopZ localization is not through the regulation of PopZ expression nor via the indirect regulation of TipN or ParA localization. (A) Western blot analysis of PopZ expression in C. crescentus mutation strains. The C. crescentus cells expressing a sole copy of *mcherry-popZ* were synchronized and transferred to the liquid M2G medium. The same cell mass was taken from different mutation strains at 1.5 h and 2 h, respectively. The expression level of mCherry-PopZ was analyzed by Western blotting using an anti-mCherry antibody. (B) Detection of the total proteins in cells. Total proteins from the samples in panel A were detected by SDS-PAGE. (C) Quantification in C. crescentus mutation strains reveals that the expression of PopZ is not regulated by PodJ, ZitP, or TipN. The grayscale values of Western blot and SDS-PAGE results were quantified with FIJI/ImageJ (49). The corresponding expression levels of mCherry-PopZ in C. crescentus strains were normalized by Western blot values against the total signal of SDS-PAGE lanes, respectively. Two independent experiments were performed and statistical analyses were executed using GraphPad Prism 5.0. Statistically significant differences were determined using Welch’s unpaired *t* test. ns, *P ≥ *0.05. (D) Quantitative analysis of mCherry-PopZ signal at the old pole, cell body, and the new pole in Δ*podJ* and the wild-type cells. Each experiment was performed in three biological replicates. A total of 33 cells (*n*) were calculated for each sample set. Corresponding to Fig. 2A. (E and F) The subcellular localization of TipN and ParA is not modulated by PodJ. The *tipN* and *parA* gene were integrated into the chromosome, respectively, to observe their subcellular localization changes after deletion of *podJ* in C. crescentus. The fluorescent tags were integrated at the 3′ end of the *tipN* and *parA* gene, respectively. Quantification of protein localization patterns is shown at the bottom. (G) The subcellular localization of YFP-ZitP_1-43_ may be regulated by PopZ instead of PodJ. Fluorescently labeled *zitP*_1-43_ (5′-end labeling) was used as described in reference 22. The majority of YFP-ZitP_1-43_ localization was changed from bipolar to old polar after deletion of *podJ*, which is likely the result of the loss of bipolar PopZ, rather than the cause (22). All scale bars = 1 μm. Download FIG S2, PDF file, 0.6 MB. Copyright © 2023 Lu et al. 2023 Lu et al. https://creativecommons.org/licenses/by/4.0/ This content is distributed under the terms of the Creative Commons Attribution 4.0 International license.

### PodJ interacts with PopZ directly through the coiled-coil 4-6 domain.

**FIG 3:**
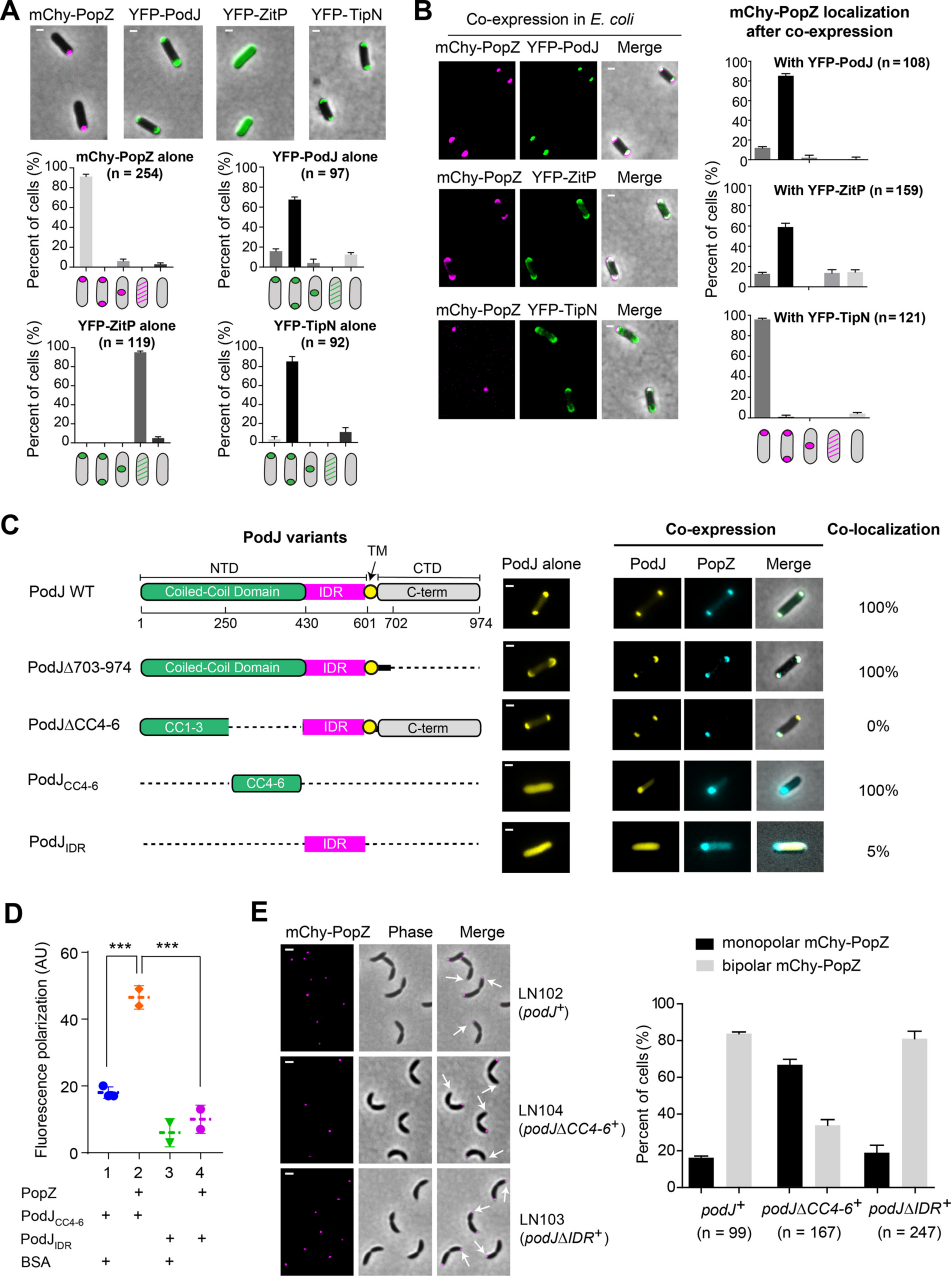
PopZ binds directly to the coiled-coil 4-6 region (CC4-6) of PodJ. (A) Heterologous expression of mCherry-PopZ alone exhibits a monopolar localization pattern, while expression of YFP-PodJ alone exhibits a bipolar localization pattern in E. coli. YFP-ZitP and YFP-TipN exhibit diffuse and bipolar localization patterns, respectively. The quantification of cells with different localization patterns is shown at the bottom. (B) PopZ interacts with PodJ and ZitP in E. coli. The mCherry-PopZ protein was coexpressed with YFP-PodJ, YFP-ZitP, or YFP-TipN. Quantitative analyses revealed that both YFP-PodJ and YFP-ZitP trigger the bipolar accumulation of mCherry-PopZ in E. coli, whereas YFP-TipN does not. (C) CC4-6 is shown as the interaction region between PodJ and PopZ in E. coli. Six coiled-coil domains (CC1-6) were predicted at the N-terminal of PodJ. Schematic diagrams of the truncated PodJ proteins are shown on the left. The colocalization values between PopZ and PodJ variants were calculated as a percentage (colocalized cell numbers/total cell numbers) and are shown on the right. When coexpressed with PopZ, for variants of PodJ that when expressed alone exhibited bipolar accumulation, we calculated the percentage of cells that also had bipolar PopZ foci and that for variants of PodJ that when expressed alone exhibited diffuse localization, we calculated the percentage of cells that exhibited colocalized PodJ foci. At least 200 cells (n) were counted in each sample. AU, arbitrary units; NTD, N-terminal domain; IDR, intrinsically disordered region; CTD, C-terminal domain; TM, transmembrane domain. (D) PopZ binds specifically to the CC4-6 domain of PodJ *in vitro*. A fluorescence polarization binding assay was performed using 100 nM BODIPY dye-labeled PodJ_IDR_ or PodJ_CC4-6_ mixed with 10 μM PopZ. Bovine serum albumin was used as a negative control. Three independent experiments were analyzed. (E) CC4-6 is responsible for the new pole accumulation of PopZ in C. crescentus. In the predivisional cell stage after synchronization, the subcellular localization of mCherry-PopZ was observed in the LN102 (NA1000 Δ*podJ*, P*_podJ_*-*podJ*, P*_popZ_-mcherry-popZ*, and P*_parB_-cfp-parB*), LN103 (NA1000 Δ*podJ*, P*_podJ_*-*podJΔIDR*, P*_popZ_-mcherry-popZ*, and P*_parB_-cfp-parB*), and LN104 (NA1000 Δ*podJ*, P*_podJ_*-*podJΔCC4-6*, P*_popZ_-mcherry-popZ*, and P*_parB_-cfp-parB*) strains. The observation of the stalk at the cell pole is considered the old cell pole. White arrows indicate the new cell poles in LN104. Quantification of the cells with different mCherry-PopZ localization patterns is shown on the right. Each experiment was performed in three biological replicates. All scale bars = 1 μm.

The gammaproteobacterium E. coli is highly divergent from the alphaproteobacterium C. crescentus and has been extensively used as a cell platform for testing C. crescentus protein-protein interactions (15, 17). Heterologous expression of mCherry-PopZ or YFP-PodJ exhibited monopolar and bipolar localization patterns in E. coli, respectively (19, 27). However, mCherry-PopZ localized in a bipolar pattern when coexpressed with YFP-PodJ (Fig. 3A and B). In contrast, PopZ did not follow the localization pattern of either TipN or ZitP in the coexpression assay (Fig. 3B), further supporting the primary role of PodJ in triggering the new pole accumulation of PopZ.

Given the regulatory effect of PodJ upon PopZ accumulation, we analyzed the coexpression of a small library of PodJ truncations with PopZ in E. coli to identify the regions within PodJ that are required for the interactions (Fig. 3C and Fig. S3). We considered the following outcomes as indicative of interactions between PodJ variants and PopZ: (i) the two proteins were 100% colocalized, and (ii) the localization pattern changed for either of the two proteins after coexpression. Failure to meet either criterion meant that the PodJ variant did not interact with PopZ or the interaction was uncertain. The results showed that deletion of the C-terminal periplasmic domain or the intrinsically disordered region (IDR) in PodJ did not affect the PodJ-PopZ interaction. In contrast, deletion of the coiled-coil 4-6 region (CC4-6) disrupted PodJ colocalization with PopZ (Fig. 3C and Fig. S3). Expression of mCherry-PodJ_CC4-6_ alone showed diffusion throughout the cytoplasm in E. coli. However, colocalization of mCherry-PodJ_CC4-6_ and CFP-PopZ was observed when coexpressed (Fig. 3C). These data indicate that CC4-6 of PodJ could be the domain for interaction with PopZ. To support this conclusion, a fluorescence polarization assay was employed to detect the binding interaction between the purified PopZ and PodJ variants (see Materials and Methods). In this assay, 16 μM PopZ and 100 nM fluorescently labeled BODIPY-PodJ_CC4-6_ or BODIPY-PodJ_IDR_ were used for incubation at room temperature for 2 h. The results demonstrated that PopZ bound to PodJ_CC4-6_ directly but did not bind to PodJ_IDR_, suggesting PodJ interacts with PopZ through the CC4-6 domain *in vitro* (Fig. 3D).

10.1128/mbio.03218-22.3 FIG S3 Identification of the N terminus of PodJ as the possible interaction region with PopZ. The schematic diagrams of truncated PodJ proteins are shown on the left. The cells with colocalized PopZ and PodJ variants were calculated and are shown on the right. At least 200 cells were counted in each sample. TM, transmembrane domain; IDR, intrinsically disordered region; TPR, tandem tetratricopeptide repeats; PG, peptidoglycan binding domain; PED, intrinsically disordered region in PopZ (PopZ_24-102_) (15). All scale bars = 1 μm. Download FIG S3, PDF file, 0.2 MB. Copyright © 2023 Lu et al. 2023 Lu et al. https://creativecommons.org/licenses/by/4.0/ This content is distributed under the terms of the Creative Commons Attribution 4.0 International license.

To confirm the interaction site between PodJ and PopZ in C. crescentus, two complementary strains were constructed under the endogenous *podJ* promoter (*podJΔCC4-6^+^* and *podJΔIDR^+^*) based on Δ*podJ* to monitor the new pole accumulation of mCherry-PopZ (Fig. 3E). PodJΔCC4-6 and PodJΔIDR proteins accumulated at the new cell pole similarly to the full-length PodJ in C. crescentus (Fig. S4A). However, only 30% of the cells harbor the bipolar mCherry-PopZ in the *podJΔCC4-6* complementary strain, much less than the observations in wild-type and full-length *podJ* complementary strains (~80% of the cells). As a control, more than 80% of *podJΔIDR* complementary cells were shown to harbor the bipolar mCherry-PopZ (Fig. 3E). Together, these results suggest that CC4-6 in PodJ is the region that essential for the interaction with PopZ both *in vitro* and *in vivo*.

10.1128/mbio.03218-22.4 FIG S4 The PodJ variant that lack of CC4-6 is able to accumulate but causes a higher mobility of CFP-ParB at the new cell pole. (A) The PodJ variant is able to accumulate at the new cell pole whereas PopZ is unable to accumulate at the new cell pole when CC4-6 of PodJ is lacking. The sfGFP-PodJ variants and mCherry-PopZ were expressed as the sole copy in C. crescentus chromosome under the P*_xyl_* or P*_van_* promoter. Relatively low concentration of inducer (0.003% xylose or 0.05 mM vanillate) was used for 3 h in this assay. Quantitative analyses were performed for the signal intensities of PodJ variants and PopZ along the cell lengths. At least 20 cells (*n*) were calculated for each test set. Data are normalized with the highest intensity as 100% in cells. (B) The CFP-ParB focus has a higher mobility at the new pole of C. crescentus with PodJ variant that lack of CC4-6. The CFP-ParB, mCherry-PopZ, and PodJΔCC4-6 were expressed as the sole copy under the endogenous promoter, respectively, in wild-type C. crescentus JP468 (NA1000, P*_parB_*-*cfp*-*parB*, and P*_popZ_*-*mcherry*-*popZ*) or in LN104 (NA1000 Δ*podJ*, P*_parB_*-*cfp*-*parB*, P*_popZ_*-*mcherry*-*popZ*, and P*_podJ_*-*podJΔCC4-6*). Analysis of the average displacements of CFP-ParB foci to the cell poles is shown. Each point refers to an average value of displacements for a focus during 22 minutes with an interval of 2 minutes. At least 40 CFP-ParB foci were calculated for each sample. Statistically significant differences were determined using Welch’s unpaired *t* test. ***, *P < *0.001. Download FIG S4, PDF file, 0.3 MB. Copyright © 2023 Lu et al. 2023 Lu et al. https://creativecommons.org/licenses/by/4.0/ This content is distributed under the terms of the Creative Commons Attribution 4.0 International license.

Additionally, the regions within PopZ that are required for the interaction with PodJ were analyzed. The E. coli coexpression results showed that deletion of either H1-, IDR-, or H3-H4-containing domains (except the H2-containing domain) in PopZ could disrupt the PodJ-PopZ interaction, indicating a multivalent binding between PopZ and PodJ (Fig. S5). This interaction is different from the PopZ-ParA or PopZ-ZitP interaction, in which only the N-terminal H1 region of PopZ was required (22, 23).

10.1128/mbio.03218-22.5 FIG S5 Identification of the domains of PopZ interaction with PodJ by heterologous coexpression experiments. The PopZ variants were expressed alone or coexpressed with PodJ in E. coli to observe their changes of subcellular localization. Colocalization results showed that PopZ_H2 domain is not responsible for the interaction with PodJ. Scale bars = 1 μm. Download FIG S5, PDF file, 0.07 MB. Copyright © 2023 Lu et al. 2023 Lu et al. https://creativecommons.org/licenses/by/4.0/ This content is distributed under the terms of the Creative Commons Attribution 4.0 International license.

### Deletion of *podJ* impairs PopZ-mediated chromosome segregation.

**FIG 4:**
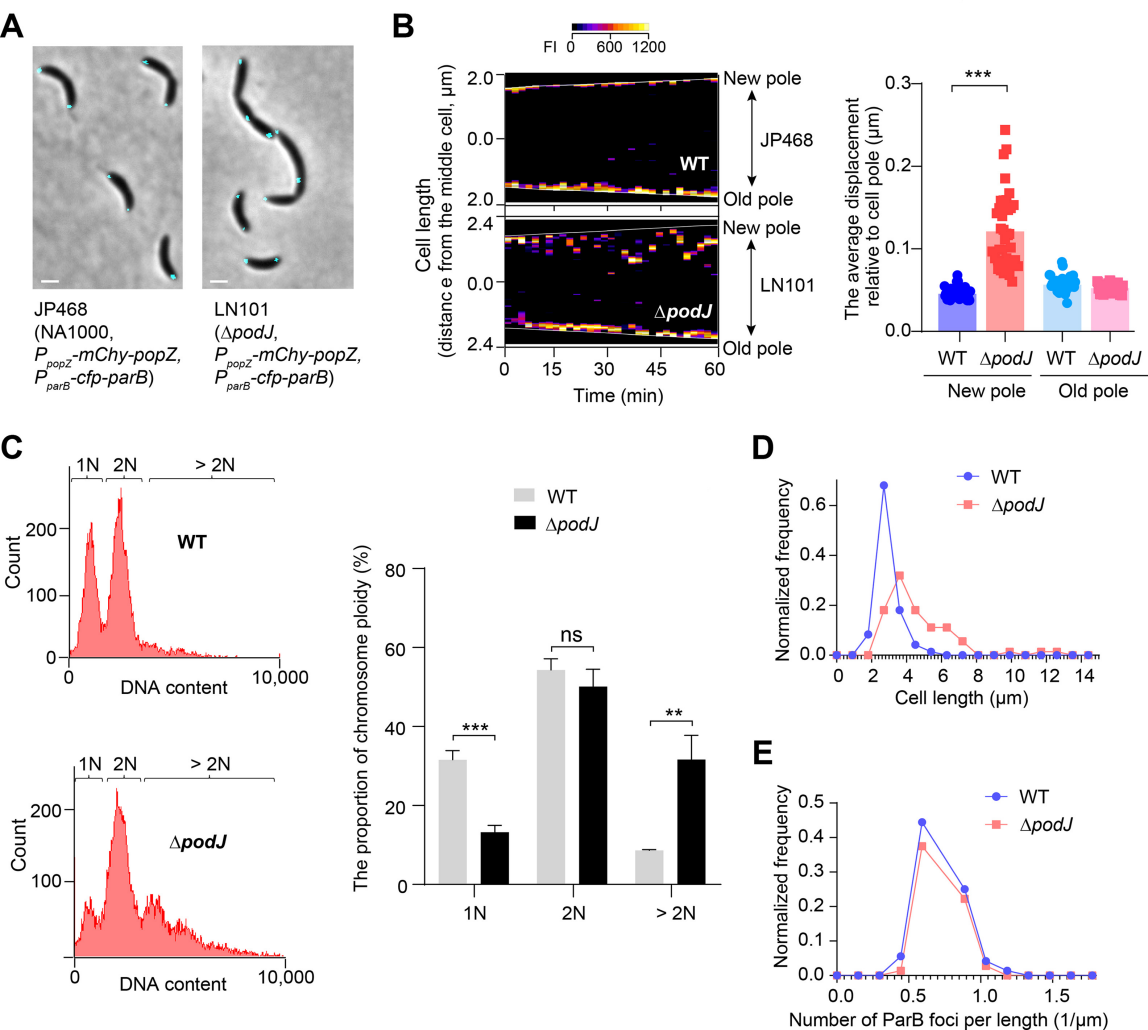
C. crescentus cells that lack PodJ exhibit chromosome segregation defects. (A) Deletion of *podJ* shows different morphology and different CFP-ParB localization compared to wild-type C. crescentus. Microscopy images of JP468 (wild type [WT]) and LN110 (Δ*podJ*) cells after cultivation in liquid PYE medium for 2 days. All scale bars = 1 μm. (B) The CFP-ParB focus has higher mobility at the new pole of Δ*podJ* than in wild-type cells. Kymographs of CFP-ParB fluorescence intensity (FI) along the cell length were recorded in the representative WT and Δ*podJ* cells over time. Images were acquired every 2 min for cells on the PYE pad. Analysis of the average displacements of ParB foci to the cell poles in WT versus Δ*podJ* cells is shown on the right. Each point refers to an average value of displacements for a focus during 36 min. At least 40 ParB foci were calculated for each sample. (C) Flow cytometry analyses suggest that deletion of *podJ* results in an increased proportion of > 2N cells and a decreased proportion of 1N cells (N indicates the chromosome ploidy in C. crescentus cells). Each experiment was performed in three biological replicates. A minimum of 10,000 cells were counted per experiment. The results are quantified and shown on the right. (D) C. crescentus cells that lack PodJ exhibit elongated cell lengths. Histograms of cell lengths for the indicated strains are shown, with frequency plotted versus cell length. At least 70 cells were calculated in each sample. (E) The C. crescentus cells lacking PodJ display comparable numbers of ParB foci per unit cell length. The normalized frequency of ParB foci number per micrometer of cell length is plotted for the indicated strains. The distributions of ParB foci per normalized cell length are similar between the wild-type and Δ*podJ* cells. ***, *P < *0.001; **, *P < *0.01; ns, *P ≥ *0.05 by Welch’s unpaired *t* test.

In C. crescentus, the partitioning and positioning of the ParB-*parS* centromere are reliant on PopZ, as PopZ tightly tethers the centromere at the cell poles (18, 19). Given that PodJ regulates the new pole accumulation of PopZ (Fig. 2 and Fig. 3E), we asked whether PodJ would affect the chromosome segregation of C. crescentus. Fluorescence microscopy for the stationary-phase cells showed that deletion of *podJ* resulted in filamentous phenotype while CFP-ParB was able to localize to the surrounding area of the Δ*podJ* new pole (Fig. 4A). However, the relative displacements of CFP-ParB foci were greater in Δ*podJ* than in wild-type cells at the new pole by both single-cell analysis and population quantification. On the contrary, no noticeable difference at the old poles was observed (Fig. 4B). The increase of CFP-ParB mobility at the new pole was also monitored in *podJΔCC4-6* cells (Fig. S4B). These results are similar to the defects in centromere positioning caused by a lack of PopZ-ParB interaction (18, 37), indicating that PodJ regulates the centromere tethering indirectly and is likely through the interaction with PopZ at the new cell pole.

Flow cytometry analysis for Δ*podJ* revealed an increase in cell count for cells harboring more than two copies of chromosomes (>2N) and a decrease in cell count for cells harboring the sole copy of chromosome (1N), compared with wild-type C. crescentus (Fig. 4C). This increased chromosome copy number in cell population indicates that PodJ may also regulate centromere partitioning. Indeed, we observed that the deletion of *podJ* resulted in elongated cell-length defects and, moreover, showed more than two foci of CFP-ParB in cells (Fig. 4D). However, quantitative analyses of the whole-cell population demonstrated that the distributions of CFP-ParB foci per normalized cell length were nearly identical between Δ*podJ* and wild-type C. crescentus (Fig. 4E). Given that similar phenotypes have been reported in *popZ* mutant cells (23), we speculated that the PodJ effect on centromere partitioning may be indirect through PopZ regulation, leading to the ongoing DNA replication and cell growth without proper cell division. Hence, these results suggest that the lack of PodJ-PopZ interaction impairs the strict coupling of one round of DNA replication per cell division in C. crescentus.

### PodJ-PopZ interaction is conserved among alphaproteobacteria.

**FIG 5:**
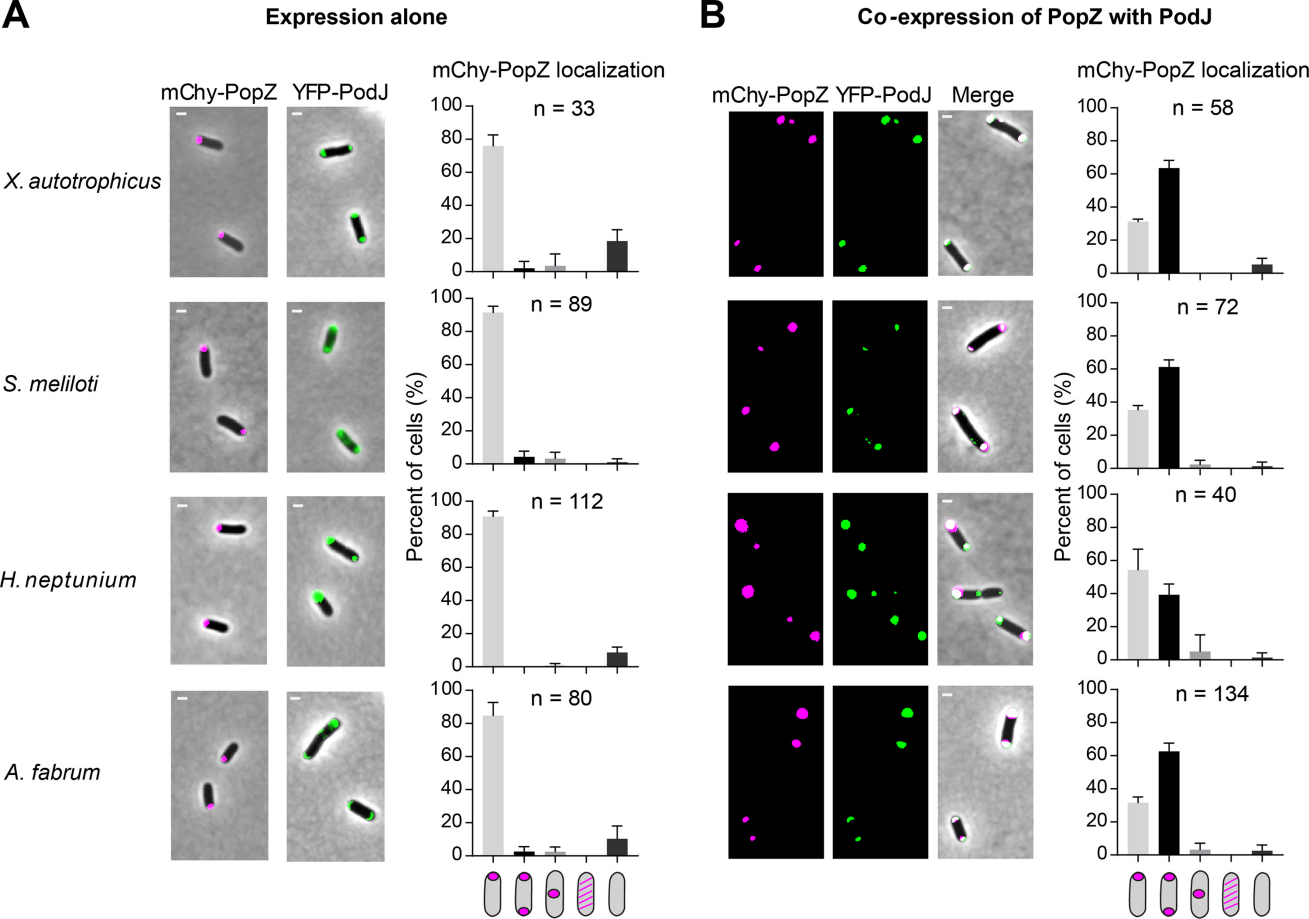
The interaction between PopZ and PodJ is conserved among alphaproteobacteria. The orthologs of C. crescentus PodJ and PopZ were selected from Xanthobacter autotrophicus, Sinorhizobium meliloti, Hyphomonas neptunium, and *Agrobacterium fabrum* and expressed alone (A) or coexpressed (B) in E. coli. All mCherry-PopZ tend to accumulate at one cell pole when expressed alone. However, the coexpression of mCherry-PopZ with YFP-PodJ results in colocalized PopZ-PodJ localization. Histograms on the right show the quantification of mCherry-PopZ localization patterns. Each experiment was performed in four biological replicates. All scale bars = 1 μm. The corresponding PopZ and PodJ in alphaproteobacteria were obtained on the NCBI database using the BLASTP program with C. crescentus PopZ and PodJ as the query sequences, respectively. *X. autotrophicus* PopZ, Xaut_4236; *X. autotrophicus* PodJ, Xaut_3064; S. meliloti PopZ, SMc02081; S. meliloti PodJ, SMc02230; *H. neptunium* PopZ, HNE_1677; *H. neptunium* PodJ, HNE_0666; *A. fabrum* PopZ, Atu1720; *A. fabrum* PodJ, Atu0499.

Orthologous gene analysis revealed that a subset of alphaproteobacteria encodes both PopZ and PodJ scaffolding proteins (Fig. S6A and B). Notably, in Agrobacterium fabrum, previous studies have shown a strong genetic interaction between PodJ and PopZ (38–40). However, it remains unclear if PodJ and PopZ interact directly or indirectly in these alphaproteobacteria. To test this, we heterologously expressed the orthologs of PodJ and PopZ in E. coli from randomly selected alphaproteobacteria, including Xanthobacter autotrophicus, Sinorhizobium meliloti, Hyphomonas neptunium, and *A. fabrum* (Fig. 5). Each PopZ ortholog accumulated in a monopolar pattern when expressed alone, similar to C. crescentus PopZ. Each PodJ ortholog accumulated at the cell poles, but compared to C. crescentus PodJ, the orthologs displayed heterogeneity in their subcellular localization patterns (Fig. 5A and Fig. S6C). However, in each case, we observed the colocalization and bipolarization of PopZ when coexpressed with PodJ (Fig. 5B). These results support direct interactions between PopZ and PodJ in a subset of alphaproteobacterial cells. Although the whole sequence similarities were not very high (22% to 35%) in the PodJ orthologs, the coiled-coli domains were predicted in each of these PodJ proteins, indicating a possible conserved mechanism through these regions to interact with PopZ among alphaproteobacteria (Fig. S6B).

10.1128/mbio.03218-22.6 FIG S6 Systematic analyses of PodJ and PopZ orthologs in α-proteobacteria. (A) Protein orthologous analyses reveals that PopZ and PodJ are encoded in a subset of α-proteobacteria. The corresponding orthologs in α-proteobacteria were obtained through BLASTP program using C. crescentus proteins as the query sequences, respectively (identity >20%; coverage >30%). Phylogenetic tree was built by MEGA X (59) using 21 amino acid sequences of CckA with Neighbor-Joining method (bootstrap: 1,000). Black dots indicate the presence of corresponding orthologs. (B) Domain analyses of PopZ and PodJ orthologs in selected α-proteobacteria. Domains of PopZ and PodJ orthologs were annotated by alignments of protein sequences. Protein identities that higher than 20% (compared with C. crescentus PopZ or PodJ ortholog) were marked above the schematic domains. Prediction of coiled-coil regions in PopZ and PodJ orthologs was achieved using DeepCoil2 (60). Prediction of intrinsically disordered regions (IDR) was achieved using IUPred3 (55). TM, transmembrane domain. (C) Quantitative analyses of YFP-PodJ localization patterns in E. coli. At least 47 cells (*n*) were calculated in each sample. The corresponding PodJ in α-proteobacteria were obtained through BLASTP program using C. crescentus PodJ as the query sequences in the KEGG database. C. crescentus PodJ, CCNA_02125; *X. autotrophicus* PodJ, Xaut_3064; S. meliloti PodJ, SMc02230; *H. neptunium* PodJ, HNE_0666; *A. fabrum* PodJ, Atu0499. Download FIG S6, PDF file, 0.4 MB. Copyright © 2023 Lu et al. 2023 Lu et al. https://creativecommons.org/licenses/by/4.0/ This content is distributed under the terms of the Creative Commons Attribution 4.0 International license.

## DISCUSSION

**FIG 6:**
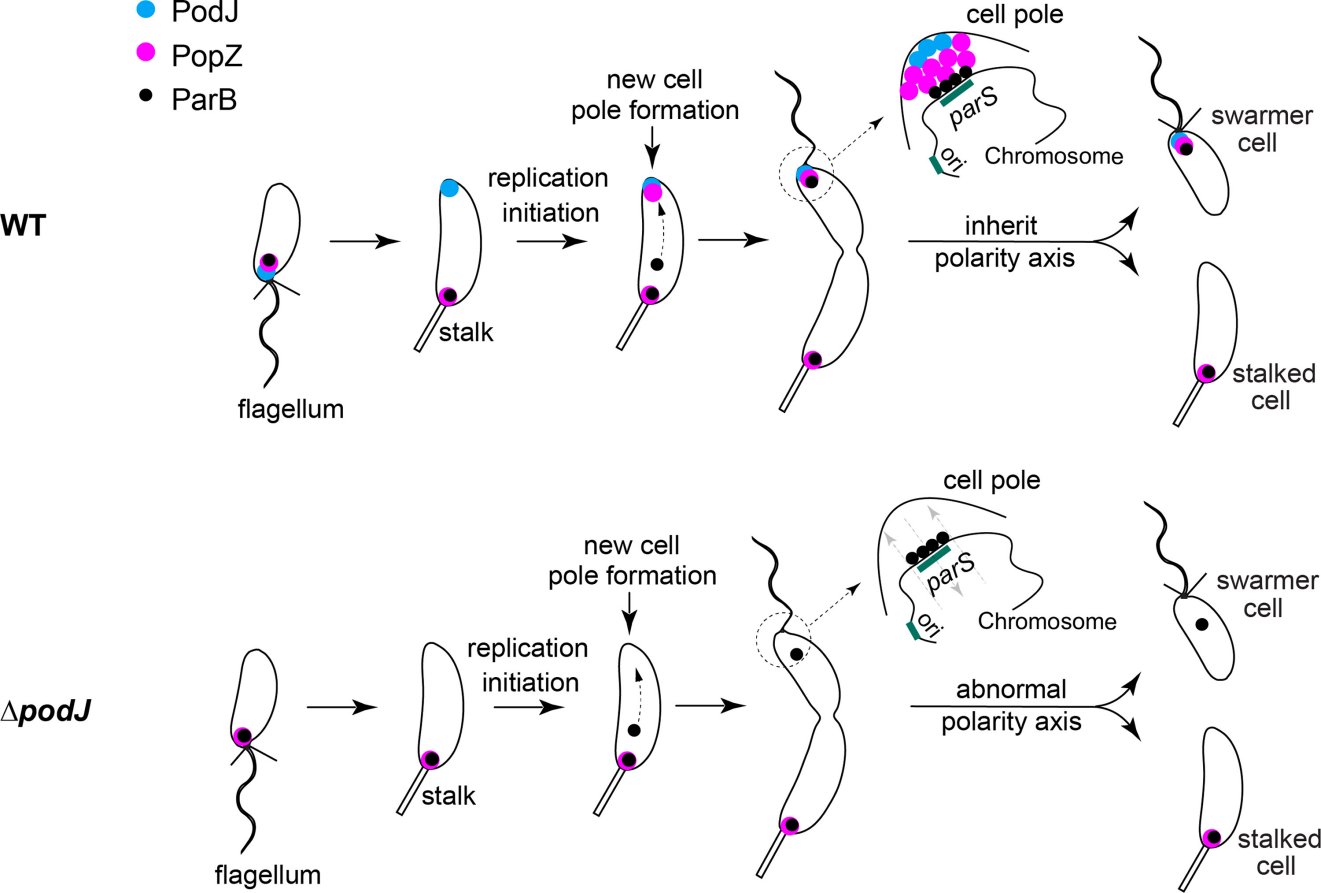
PodJ-PopZ interaction regulates cell polarity development in C. crescentus. In C. crescentus, scaffold-scaffold interactions between PopZ and PodJ facilitate monopolar to bipolar accumulation of PopZ during the predivisional cell stage. PodJ ensures timely accumulation of the polar PopZ, and the PodJ-PopZ interaction is essential for anchoring the ParB-*parS* centromere at the new pole. After cell division, the daughter cells inherit distinct polarity signaling hubs. In cells lacking PodJ, PopZ fails to accumulate at the new pole at the appropriate time. Disruption of the PodJ-PopZ interaction impairs the PopZ-mediated tethering of the ParB-*parS* centromere at the new cell pole. Moreover, abnormal cells with elongated cell lengths and increased chromosome copy numbers are generated, possibly due to the improper cell division and the ongoing DNA replication and cell growth (23).

A key event in cell polarization is forming a new polar signaling hub compositionally and functionally distinct from the opposite old cell pole. Both scaffold proteins PopZ and PodJ have been shown to be the organizers of cell-pole signaling hubs in C. crescentus (15, 21, 27, 28). However, the spatiotemporal regulation of these scaffolds on cell polarity development remains largely unknown. Here, we report a direct and conserved interaction between PopZ and PodJ scaffolds, which is a significant event for triggering monopolar to bipolar accumulation of PopZ (Fig. 6). The primary regulatory role of PodJ was demonstrated in parallel by comparing it with other PopZ regulators, such as ZitP (22) and TipN (17). The CC4-6 domain in PodJ was responsible for the PodJ-PopZ interaction both *in vitro* and *in vivo*. PodJ ensured timely accumulation of the polar PopZ and inheritance of the polarity axis. Disruption of the PodJ-PopZ interaction impaired PopZ-mediated chromosome segregation and may lead to a decoupling of DNA replication from cell division during the cell cycle (Fig. 6).

Utilizing a fluorescent timer approach, we have demonstrated that the newly synthesized PopZ was enriched at the new cell pole while the old PopZ was retained mainly at the old cell pole (Fig. 1B to D). In support of this finding, the transition of PopZ from monopolar to bipolar location pattern was observed along with an increase of *popZ* transcription during the cell cycle (32). The little migration of PopZ from the old cell pole to new cell pole may be because it is a self-assembled protein that tends to oligomerization and stable binding together (17, 21). Given that PopZ recruits distinct signaling proteins at opposite poles (15, 37), we speculate that the accumulation of PopZ with different ages may play a role in preventing the homogenization of the two polar PopZ clients.

In the current study, PodJ was shown to be the primary regulator that promoted the new pole accumulation of PopZ in C. crescentus (Fig. 2 and 3). However, critical roles in the subcellular regulation of PopZ have also been characterized for ZitP (22) and TipN (17). Indeed, our study has shown that in the absence of PodJ, PopZ can still accumulate as foci at the new cell poles in ~20% of predivisional cells (Fig. 2). These observations indicate that PodJ is sufficient but may not be essential for PopZ new pole accumulation. Some redundant factors such as ZitP and TipN could be functional in this process (17, 22). Nevertheless, PodJ, ZitP, and TipN promote the PopZ bipolarization in very different ways. Although ZitP can interact with PopZ directly, it was shown to have the lowest effect on PopZ bipolarization among these factors, possibly owing to that ZitP was unable to self-assemble *in vivo* (Fig. 3A). The localization of ZitP was dependent on PopZ instead (Fig. 2 and Fig. 3). TipN had a moderate effect on PopZ bipolarization compared to ZitP (Fig. 2B, C). However, the effect may be implemented through other factors, such as ParA (15), because no direct interaction was detected between TipN and PopZ (Fig. 3). In contrast, PodJ played a primary role in promoting bipolar PopZ both in C. crescentus and in E. coli (Fig. 2 and Fig. 3).

The PodJ effect upon PopZ new pole accumulation may not be through the regulation of *popZ* expression (Fig. S2A to C) nor via the indirect reduction of *zitP*, *tipN*, or *parA* transcription, the latter of which was demonstrated by the RNAseq analysis using a 750× Illumina sequencing (see Materials and Methods and Fig. S7). In total, 316 genes were differentially expressed after deletion of *podJ*, including 135 upregulated genes and 181 downregulated genes but not containing the factors mentioned above (Fig. S7 and Table S2). In contrast to that, PodJ’s impact on PopZ new pole accumulation could occur through a direct interaction with PopZ using the CC4-6 domain in C. crescentus (Fig. 3). To ask where the extra PopZ protein that used to be at the new pole is in Δ*podJ*, we performed a quantitative analysis to measure the mCherry-PopZ signal at the old pole, cell body and the new pole in Δ*podJ* and the wild-type cells. Comparing the proportions of these signals indicated that the extra PopZ protein that used to be at the new pole is mostly accumulated at the old pole and partially free in the cytoplasm in Δ*podJ* (Fig. S2D). On the other hand, by monitoring the labeled TipN and ParA proteins in Δ*podJ* cells, possible indirect regulation of their subcellular localization by PodJ was excluded (Fig. S2E and F). Although the majority of YFP-ZitP_1-43_ localization was changed from bipolar to old-polar after deletion of *podJ* (Fig. S2G), the result of which is likely the consequence of the loss of bipolar PopZ rather than the cause. Nevertheless, these results suggest that the PodJ regulation upon PopZ subcellular accumulation is direct and significant (Fig. 2 and 4).

10.1128/mbio.03218-22.7 FIG S7 Transcriptome analysis after deletion of *podJ* gene. (A) Volcano plot of the differentially expressed genes between Δ*podJ* and JP468 strains. A total of 6 samples with 3 biological repeats were used for RNA extraction and transcriptome sequencing in 1.5 h after synchronization. The changes in gene transcriptions were displayed after deletion of *podJ* in JP468 strain. Magenta dots represent the significantly upregulated genes, green dots represent the significantly downregulated genes, and gray dots indicate the nonsignificance of difference in gene transcription between the two strains. The numbers of differentially regulated genes in Δ*podJ* strain were summarized as below. For more details, please see Table S2. (B) The transcription levels of *popZ*, *tipN*, *parA*, and *parB* are not affected by the deletion of *podJ.* The results indicate that the promotion of bipolar PopZ by PodJ was not through the regulation of PopZ expression, nor via the indirect regulation of expression of TipN or ParA. The transcription level of *zitP* is even slightly increased in Δ*podJ*, indicating the promotion of bipolar PopZ by PodJ was not through the indirect regulation of ZitP expression either. FPKM, fragments per kilobase of exon model per million mapped fragments. ****, *P < *0.0001; **, *P < *0.01; *, *P < *0.05; ns, *P ≥ *0.05 by two-tailed paired *t* test. Download FIG S7, PDF file, 0.1 MB. Copyright © 2023 Lu et al. 2023 Lu et al. https://creativecommons.org/licenses/by/4.0/ This content is distributed under the terms of the Creative Commons Attribution 4.0 International license.

10.1128/mbio.03218-22.9 TABLE S2 The genes that are differentially regulated in the podJ deletion strain. Download Table S2, XLSX file, 0.1 MB. Copyright © 2023 Lu et al. 2023 Lu et al. https://creativecommons.org/licenses/by/4.0/ This content is distributed under the terms of the Creative Commons Attribution 4.0 International license.

The PodJ-PopZ interaction is conserved while the subcellular localization patterns of PodJ and PopZ may be diverse in different alphaproteobacterial organisms (38, 40–43) (Fig. 5). In *A. fabrum*, a genetic interaction between PodJ and PopZ was observed (38, 40). However, its polarity was inverted compared to C. crescentus, since PopZ residues exclusively at the new cell pole while PodJ occupies the old cell pole (40). In addition, another subset of alphaproteobacteria species encode the PopZ scaffold and the CckA histidine kinase, but their genomes contain no clear homologs of the PodJ encoding genes (Fig. S6A). These variations in conservation suggest that the PopZ-PodJ polarity network may have been rewired to support diverse modes of cell development (44).

Recently, biomolecular condensation has emerged as a general principle for cell polarity development (4, 21, 45). The scaffold proteins PopZ, PodJ, and SpmX have been shown to form matrixes or biomolecular condensates at the cell poles (21, 27, 46). Phase separation is involved in the protein assembly of PopZ and SpmX and regulates the interaction at the old cell pole (21, 46). Our recently published data also suggest that PodJ phase separation plays a critical role in new cell pole assembly (47). Unlike scaffold-client recruitment, scaffold-scaffold interaction may provide an underlying infrastructure for cell polarity development (5). Key questions remain as to the factors that promote the coassembly of these scaffolds and the compositional control of biomolecular condensates at the opposite cell poles. A system-level understanding of the assembly of these biomolecular condensates will contribute to the cell polarity research field.

## MATERIALS AND METHODS

### Bacterial strains, plasmids, and culture conditions.

The strains, plasmids, and primers used in this study are summarized in Table S3. Recombinant C. crescentus strains were obtained using either homologous recombination (48) or the CRISPR/Cas9 genome editing method. Recombinant E. coli strains were obtained using the standard clone method. Details regarding the constructions of these strains are described below. As indicated, the wild-type C. crescentus strain NA1000 and its derivatives were grown aerobically at 30°C in peptone yeast extract (PYE) rich medium or M2G minimal medium. E. coli strains were grown aerobically at 37°C in Luria-Bertani (LB) medium.

10.1128/mbio.03218-22.10 TABLE S3 Plasmids, strains, and oligonucleotides used in this study. Download Table S3, PDF file, 0.3 MB. Copyright © 2023 Lu et al. 2023 Lu et al. https://creativecommons.org/licenses/by/4.0/ This content is distributed under the terms of the Creative Commons Attribution 4.0 International license.

Antibiotics were used when required at the following concentrations (liquid/solid media for C. crescentus; liquid/solid media for E. coli; μg/mL): kanamycin (5/25; 50/50), chloramphenicol (1/5; 30/30), spectinomycin (not applicable; 50/50), and ampicillin (not applicable; 100/100). When required, gene expression was induced in C. crescentus with either 0.03% (wt/vol) xylose or 0.5 mM vanillate for 3 h unless otherwise stated. Gene expression in E. coli was induced by adding 0.1 to 0.5 mM isopropyl β-d-1-thiogalactopyranoside (IPTG) or 1 to 10 mM l-arabinose for 2 h. All reagents used in this study were purchased from Sigma-Aldrich unless otherwise stated.

### Deletion of *podJ*, *zitP*, and *tipN* in C. crescentus.

JP468 (NA1000, P*_popZ_-mcherry-popZ*, P*_parB_-cfp-parB*) was used in this study based on previous work showing that fusion proteins of mCherry-PopZ and CFP-ParB do not affect the cell growth or cell cycle in C. crescentus (23). In-frame deletion of the *podJ*, *zitP*, or *tipN* gene in the C. crescentus JP468 strain was achieved using a modified CRISPR-Cas9 editing system. In brief, an all-in-one plasmid containing homologous arms, guide RNA, and codon-optimized Cas9 was constructed and electroporated into the JP468 strain. The correct clones were checked by PCR and further confirmed by DNA sequencing. The potential off-targets were tested using whole-genome sequencing, and no off-targets were observed.

### Construction of *podJ* complementary strains.

The promoter of the *podJ* gene (~200 bp) and the coding sequences of *podJ* or its variants *podJΔIDR* and *podJΔCC4-6* were amplified or subcloned from the wild-type C. crescentus genome. These fragments were assembled into a pXYFPN-2 vector by Gibson assembly to obtain the recombinant plasmids. Correct plasmids were then electroporated into the Δ*podJ* strain and integrated into the chromosome by DNA single-strand exchange. Positive clones were acquired by resistance marker screening and verified by PCR.

### Phase contrast, differential interference contrast, and epifluorescence microscopy.

Cells were imaged after being immobilized on a 1.5% (wt/vol) agarose pad containing the corresponding inducers when required. Phase microscopy was performed using a Nikon Eclipse T*i*2-E inverted microscope equipped with an Andor Ixon Ultra DU897 EMCCD camera and a Nikon CFI Plan-Apochromat ×100 oil objective. Differential interference contrast (DIC) microscopy was performed using the same microscope and camera but with a Nikon DIC polarizer and slider in place. The excitation source was a Lumencor SpectraX light engine. A chroma filter cube CFP/YFP/MCHRY MTD TI was used to image enhanced cyan fluorescent protein (ECFP; 465/25 M), enhanced yellow fluorescent protein (EYFP; 545/30 M), and mCherry (630/60 M). A chroma filter cube GFP was used to image EGFP and sfGFP (470/×40, 515/30 M). Images were collected and processed with Fiji/ImageJ (49) and MicrobeJ (50).

### Time-lapse fluorescence imaging of mCherry-PopZ, mCherry-sfGFP-PopZ, and mCherry-sfGFP-RpoC.

For time-lapse imaging of mCherry-PopZ in JP468, Δ*podJ*, Δ*zitP*, or Δ*tipN*, a single clone of the strain was inoculated into M2G medium at 30°C overnight. The cells were then transferred to a fresh liquid medium and grown until the mid-log phase (optical density at 600 nm [OD_600_] = 0.5 to 0.6). Swarmer cells were isolated from the culture by centrifugation (20 min at 11,000 rpm, 4°C) after mixing one volume of Percoll (GE Healthcare) (51). The synchronized swarmer cells were pipetted onto a 1.5% (wt/vol) agarose pad containing M2G medium and sealed with wax. Phase and fluorescence images were taken simultaneously at 15- or 30-min intervals for 1 to 2 cell cycles during time-lapse experiments, using a Nikon Eclipse T*i*2-E time-lapse imaging system at 30°C (~4 h).

For time-lapse imaging of mCherry-sfGFP-PopZ or mCherry-sfGFP-RpoC, a fluorescence timer cassette of *mcherry-sfgfp* was constructed and fused to *popZ* or *rpoC*. The corresponding native promoters and the coding sequences of *mcherry-sfgfp*-*popZ* or *mcherry-sfgfp*-*rpoC* were assembled into a pXYFPN-2 vector by Gibson assembly. Recombinant plasmids were then electroporated into C. crescentus NA1000 cells. The positive clones with integrated *mcherry-sfgfp*-*popZ* or *mcherry-sfgfp*-*rpoC* in the chromosome were obtained by resistance marker screening and further verified by PCR detection. The cells were synchronized, and similar time-lapse experiments were performed as described above, except using a PYE medium with kanamycin for cell cultivation. Images were collected and processed with Fiji/ImageJ (49) and MicrobeJ (50).

### Fluorescence recovery after photobleaching analysis.

Pole-specific FRAP was employed to determine the dynamics of PopZ *in vivo*. The recovery of fluorescence signal after photobleaching was considered a fast exchange of protein molecules between the polar condensates and the surrounding aqueous solution.

Nikon A1R^+^ confocal laser scanning microscope with a 100× oil immersion lens objective was used in this experiment. C. crescentus cells (NA1000, P*_popZ_-mcherry-sfgfp-popZ*) were first synchronized as described above and cultured in PYE liquid medium to the predivisional stage. The cells were then immobilized at a 1.5% (wt/vol) agarose pad containing PYE medium at 30°C for imaging. The cell pole regions with all the PopZ accumulated (diameter: 0.2 to 0.4 μm) were selected for photobleaching. The fluorescence signal within the selected regions was bleached using a 488-nm laser at 59% laser power for approximately 2 s. After photobleaching, time-lapse images were captured every 30 s for about 3 min.

For data analysis, the fluorescence intensity of selected regions before photobleaching was normalized to 100%. Taking the average fluorescent signal of three PopZ foci without bleaching as a reference, each signal intensity of the experimental group was first normalized with the signal intensity of the reference group at each time point, and the percentage changes of the fluorescence intensity after photobleaching were calculated and plotted.

### Western blot analysis.

Western blot analysis was used to detect the expression levels of mCherry-PopZ in C. crescentus JP468, Δ*podJ*, Δ*zitP*, and Δ*tipN* strains. Cells were grown in 100 mL M2G medium at 30°C until mid-log phase (OD_600_ = 0.5 to 0.6). As described above, swarmer cells were isolated from the culture with Percoll (51). The collected swarmer cells were resuspended in 10 mL of fresh M2G medium for further incubation at 30°C. The same volumes of 4 mL cells were collected at 1.5 h and 2 h after synchronization. Cells expressing mCherry-PopZ were used for Western blot analysis according to the previously described procedures (52). The antibody against mCherry (1:1,000 dilution; Cell Signaling; cat. no: 43590) and the horseradish peroxidase-conjugated goat anti-rabbit secondary antibody (1:2,000 dilution; Cell Signaling; cat. no: 7074S) were used in this study.

### Comparison of protein localizations in Δ*podJ* and the wild-type C. crescentus.

To illustrate the effect of Δ*podJ* on subcellular localization of ZitP, TipN, and ParA, the sole copy of these fluorescently labeled proteins (P*_xyl_*-*yfp*-*zitP_1-43_*, P*_tipN_*-*tipN*-*mcherry*, and P*_parA_*-*parA*-*yfp*) were expressed by integrating their genes in Δ*podJ* and the wild-type C. crescentus chromosomes, respectively.

Single clones of the correct integrations were inoculated into liquid medium PYE for overnight culture at 30°C. For genes driven by the native promoters (P*_tipN_*-*tipN*-*mcherry* and P*_parA_*-*parA*-*yfp*), cells were transferred to fresh PYE liquid medium and grown until the mid-log phase (OD_600_ = 0.5 to 0.6). For *yfp-zitP_1-43_* driven by the P*_xyl_* promoter, C. crescentus cells were transferred to fresh PYE liquid medium and grown to the early log phase (OD_600_ = 0.2), and then induced by 0.03% xylose for 2 to 3 h. Phase and fluorescence images were then taken by Nikon the Eclipse T*i*2-E microscope as described above. The localization pattern of these proteins in Δ*podJ* and the wild-type C. crescentus cells were quantitatively analyzed with Fiji/ImageJ and MicrobeJ (50).

### E. coli coexpression assay.

To determine the interaction between PopZ and PodJ or PodJ variants, E. coli BL21(DE3) was used because it does not contain any polarity proteins homologous with C. crescentus. In total, 10 PodJ variants were constructed based on domain analysis and truncation. The expression plasmids of the PodJ variants were constructed using a fluorescent tag within the pCDF vector, while fluorescent-tagged PopZ was constructed based on the pBAD vector (Table S3).

In the coexpression assay, we examined the changes in protein subcellular localization by coexpressing the two proteins in E. coli. The expression plasmids were cotransformed into E. coli BL21(DE3) by both spectinomycin and ampicillin selection. Single clones of the correct strains were inoculated into liquid LB medium at 37°C overnight. The cells were then transferred into fresh LB medium and grown until the mid-log phase (OD_600_ = 0.4 to 0.5). Both IPTG (0.1 mM) and l-arabinose (5 mM) were added into the culture to induce protein expressions for 2 to 3 h. The cells were diluted to the appropriate concentration and pipetted onto a 1.5% (wt/vol) agarose pad containing LB medium. Images were taken using a Nikon Eclipse T*i*2-E inverted microscope.

We used the following strict criteria to determine protein colocalization: (i) either of the localization patterns of the tested proteins changed after coexpression, and (ii) the two proteins were 100% colocalized in > 90% E. coli cells. Failure to meet either criterion meant that the tested proteins were not colocalized or the colocalization was uncertain in E. coli.

### Purification of PopZ and PodJ variants.

Protein expression of PopZ and PodJ variants followed the same protocol and is described in detail below for PopZ. To purify PopZ, the E. coli BL21(DE3)-containing expression plasmid was grown in 6 liters of LB medium (20 μg/mL chloramphenicol and 100 μg/mL ampicillin) at 37°C. The culture was then induced at an OD_600_ of 0.4 to 0.6 with 0.5 mM IPTG overnight at 18°C. The cells were harvested and resuspended in lysis buffer (50 mM Tris-HCl, 700 mM KCl, 20 mM imidazole, and 0.05% dextran sulfate, pH 8.0) in the presence of protease inhibitor cocktail tablets without EDTA (Roche).

The cell suspension was lysed with three passes through an EmulsiFlex-C5 cell disruptor (AVESTIN, Inc., Ottawa, Canada), and the supernatant was collected by centrifugation at 13,000 × *g* for 30 min at 4°C. The insoluble cell debris was resuspended using the recovery buffer (50 mM Tris-HCl, 1,000 mM KCl, 20 mM imidazole, and 0.05% dextran sulfate, pH 8.0), and the supernatant was collected by additional centrifugation. The combined supernatants were loaded onto a 5 mL HisTrap HP column (GE Healthcare) and purified with the ÄKTA FPLC System. After being washed with 10 volumes of wash buffer (50 mM Tris-HCl, 300 mM KCl, and 25 mM imidazole, pH 8.0), the protein was collected by elution from the system with elution buffer (50 mM Tris-HCl, 300 mM KCl, and 500 mM imidazole, pH 8.0) and concentrated to a 3 mL volume using Amicon Centrifugal Filter Units, resulting in >90% purity. PopZ protein and PodJ variants were dialyzed with a dialysis buffer containing 50 mM Tris-HCl (pH 8.0) and 300 mM KCl and then aliquoted to a small volume (100 μL) and kept frozen at −80°C until use.

### Fluorescence polarization binding assay.

A fluorescence polarization binding assay (53) was used to measure the binding and dissociation between PopZ and PodJ variants. The His-tagged recombinant proteins used in this study were expressed and purified as described above. To label PodJ_IDR_ (431 to 601 aa) and PodJ_CC4-6_ (250 to 430 aa), a cysteine (Cys) at the N terminus of each protein was cloned just after the 6× His tag. The purified proteins were labeled by mixing with 10-fold excess thiol-reactive BODIPY FL *N*-(2-aminoethyl)maleimide (Thermo Fisher; cat. no. B10250). After incubation at room temperature for 2 h, the unreacted dye was quenched with mercaptoethanol (5% final concentration). The labeled proteins were then purified via dialysis using dialysis buffer at 4°C (30 min each, 5 times) to remove the unreacted fluorescent dye.

Fluorescence polarization binding assays were performed by mixing 100 nM labeled proteins with 0, 0.25, 0.5, 1, 2, 4, 8, and 16 μM partner protein (PopZ) for 45 min to reach binding equilibrium at room temperature. Bovine serum albumin was used as the negative control. Fluorescent proteins were excited at 470 nm, and emission polarization was measured at 530 nm in a UV-vis Evol 600 spectrophotometer (Thermo Fisher). Fluorescent polarization measurements were performed in triplicate, and three independent trials were averaged with error bars representing the standard deviation.

### CFP-ParB tracking and kymograph analyses.

For tracking of CFP-ParB foci in cells, JP468 and Δ*podJ* strains expressing CFP-ParB from the native ParB promoter were grown in a PYE medium at 30°C for 2 days to reach the stationary cell stage. The cells were then immobilized at a 1.5% (wt/vol) agarose pad containing PYE medium at 30°C, and the images were recorded every 2 min at 30°C for 60 min. Kymographs of fluorescence intensity were obtained using the built-in kymograph function of MicrobeJ (50). The background signal was subtracted before the kymograph analysis. The predivisional cell that had already segregated a CFP-ParB focus to the new cell pole was analyzed as *t *= 0 min. The distance between the CFP-ParB focus and the new cell pole was calculated every 2 min. A total of 40 cells of each strain were analyzed over 18 consecutive time intervals, yielding over 700 data points.

### Flow cytometry analysis.

To determine the chromosome ploidy of C. crescentus JP468 and Δ*podJ* cells, flow cytometry analysis was performed as described previously (54) with minor changes. Briefly, a single colony of JP468 or Δ*podJ* cells was inoculated into 5 mL of PYE medium and grown at 30°C for 2 days to reach the stationary cell stage. The cells were collected by centrifugation at 6,000 × *g* for 10 min and washed once with sterile distilled water at 4°C. For cell staining, the cell pellet was resuspended in 1 mL staining buffer (70% ethanol, 10 mM Tris-HCl, 50 mM sodium citrate, and 1 mM EDTA, pH 7.2) and incubated at −20°C overnight. Cells were then treated with RNase (20 mg/mL; Sigma) at 37°C for 2 to 3 h. Samples were further stained by PicoGreen with a 1:200 dilution (Invitrogen; cat. no: P7581) and analyzed by flow cytometry (Beckman-Coulter Cytoflex S). A minimum of 10,000 cells were counted for each sample.

### Prediction of PopZ and PodJ and analysis of their interactions in alphaproteobacteria.

The orthologs of PopZ and PodJ were analyzed in alphaproteobacteria using the BLASTP program with C. crescentus PopZ and PodJ as the query sequences in the KEGG database (https://www.kegg.jp/kegg/). The probability of an intrinsically disordered region over the primary sequence of PopZ or PodJ was predicted by IUPred3 (55). The average scores of these programs were plotted against the protein sequence. In this study, we assumed the region as an intrinsic disorder with a probability >60%.

To determine the interaction between PopZ and PodJ from randomly selected alphaproteobacteria, we examined their protein subcellular localizations by coexpressing PopZ-PodJ pairs in E. coli. Images were taken using the Nikon Eclipse T*i*2-E inverted microscope. Similar analyses were performed as described above in the E. coli coexpression assay.

### RNAseq analysis.

The single clones of JP468 and Δ*podJ* cells were inoculated in 5 mL of M2G medium at 30°C overnight. The cells were transferred to a fresh liquid medium and grown until the mid-log phase (OD_600_ = 0.5 to 0.6). After cell synchronization, swarmer cells were collected and cultured. After another 1.5 h, the cell pellets were collected and flash-frozen in liquid nitrogen. According to quantitative analyses in Fig. 2C, the deletion of *podJ* had the most significant impact on the new pole accumulation of PopZ at 1.5 h, compared to that of JP468. As previously described (32), a total of 6 samples with 3 biological repeats were used for RNA extraction and transcriptome analysis.

The transcriptome was sequenced on an Illumina Hiseq 2000 platform and ~3 gigabase data were obtained for each sample. Raw data with fastq format were first processed using in-house perl scripts to remove the reads containing adapters and trimming the low-quality bases (56). The Bowtie2 program (v 2.2.6) (57) was used to perform genome mapping analysis on the filtered sequencing sequences. A DESeq2 R package (v 1.30.0) (58) based on the negative binomial distribution model was employed to analyze the differential gene transcription between JP468 and Δ*podJ* cells. The resulting *P* values were adjusted using the Benjamini and Hochberg’s approach for controlling the false discovery rates. Genes with an adjusted *P* value <0.05 and the multiple of difference ≥2 were assigned differentially expressed.

### Statistical analyses.

The GraphPad Prism 5.0 program was used to analyze all the data. Unless otherwise stated, each experiment was performed in three biological replicates. Statistically significant differences were determined using Welch’s unpaired *t* test. ****, *P < *0.0001; ***, *P < *0.001; **, *P < *0.01; *, *P < *0.05; ns, *P ≥ *0.05. Measurements are shown as mean ± standard deviations (s.d.) where appropriate.

### Data availability.

The transcriptome sequencing data sets were uploaded to the SRA database (PRJNA923736).

## References

[1] Lasker K, Mann TH, Shapiro L. 2016. An intracellular compass spatially coordinates cell cycle modules in Caulobacter crescentus. Curr Opin Microbiol 33:131–139. doi:10.1016/j.mib.2016.06.007. 27517351 PMC5069156

[2] Piroli ME, Blanchette JO, Jabbarzadeh E. 2019. Polarity as a physiological modulator of cell function. Front Biosci (Landmark Ed) 24:451–462. doi:10.2741/4728. 30468666 PMC6343491

[3] Aakre CD, Laub MT. 2012. Asymmetric cell division: a persistent issue? Dev Cell 22:235–236. doi:10.1016/j.devcel.2012.01.016. 22340488 PMC3295579

[4] Liu Z, Yang Y, Gu A, Xu J, Mao Y, Lu H, Hu W, Lei QY, Li Z, Zhang M, Cai Y, Wen W. 2020. Par complex cluster formation mediated by phase separation. Nat Commun 11:2266. doi:10.1038/s41467-020-16135-6. 32385244 PMC7211019

[5] Chau AH, Walter JM, Gerardin J, Tang C, Lim WA. 2012. Designing synthetic regulatory networks capable of self-organizing cell polarization. Cell 151:320–332. doi:10.1016/j.cell.2012.08.040. 23039994 PMC3498761

[6] Curtis PD, Brun YV. 2010. Getting in the loop: regulation of development in Caulobacter crescentus. Microbiol Mol Biol Rev 74:13–41. doi:10.1128/MMBR.00040-09. 20197497 PMC2832345

[7] Govers SK, Jacobs-Wagner C. 2020. Caulobacter crescentus: model system extraordinaire. Curr Biol 30:R1151–R1158. doi:10.1016/j.cub.2020.07.033. 33022259

[8] Ardissone S, Fumeaux C, Berge M, Beaussart A, Theraulaz L, Radhakrishnan SK, Dufrene YF, Viollier PH. 2014. Cell cycle constraints on capsulation and bacteriophage susceptibility. Elife 3:e03587. doi:10.7554/eLife.03587. 25421297 PMC4241560

[9] Lawaree E, Gillet S, Louis G, Tilquin F, Le Blastier S, Cambier P, Matroule JY. 2016. Caulobacter crescentus intrinsic dimorphism provides a prompt bimodal response to copper stress. Nat Microbiol 1:16098. doi:10.1038/nmicrobiol.2016.98. 27562256

[10] Tsokos CG, Laub MT. 2012. Polarity and cell fate asymmetry in Caulobacter crescentus. Curr Opin Microbiol 15:744–750. doi:10.1016/j.mib.2012.10.011. 23146566 PMC3587792

[11] Bergé M, Viollier PH. 2018. End-in-sight: cell polarization by the polygamic organizer PopZ. Trends in Microbiology 26:363–375. doi:10.1016/j.tim.2017.11.007. 29198650

[12] Good MC, Zalatan JG, Lim WA. 2011. Scaffold proteins: hubs for controlling the flow of cellular information. Science 332:680–686. doi:10.1126/science.1198701. 21551057 PMC3117218

[13] Lin DW, Liu Y, Lee YQ, Yang PJ, Ho CT, Hong JC, Hsiao JC, Liao DC, Liang AJ, Hung TC, Chen YC, Tu HL, Hsu CP, Huang HC. 2021. Construction of intracellular asymmetry and asymmetric division in Escherichia coli. Nat Commun 12:888. doi:10.1038/s41467-021-21135-1. 33563962 PMC7873278

[14] Perez AM, Mann TH, Lasker K, Ahrens DG, Eckart MR, Shapiro L. 2017. A localized complex of two protein oligomers controls the orientation of cell polarity. mBio 8:e02238-16. doi:10.1128/mBio.02238-16. 28246363 PMC5347347

[15] Holmes JA, Follett SE, Wang H, Meadows CP, Varga K, Bowman GR. 2016. Caulobacter PopZ forms an intrinsically disordered hub in organizing bacterial cell poles. Proc Natl Acad Sci USA 113:12490–12495. doi:10.1073/pnas.1602380113. 27791060 PMC5098656

[16] Curtis PD, Quardokus EM, Lawler ML, Guo X, Klein D, Chen JC, Arnold RJ, Brun YV. 2012. The scaffolding and signalling functions of a localization factor impact polar development. Mol Microbiol 84:712–735. doi:10.1111/j.1365-2958.2012.08055.x. 22512778 PMC3345042

[17] Laloux G, Jacobs-Wagner C. 2013. Spatiotemporal control of PopZ localization through cell cycle-coupled multimerization. J Cell Biol 201:827–841. doi:10.1083/jcb.201303036. 23751494 PMC3678156

[18] Bowman GR, Comolli LR, Zhu J, Eckart M, Koenig M, Downing KH, Moerner WE, Earnest T, Shapiro L. 2008. A polymeric protein anchors the chromosomal origin/ParB complex at a bacterial cell pole. Cell 134:945–955. doi:10.1016/j.cell.2008.07.015. 18805088 PMC2745220

[19] Ebersbach G, Briegel A, Jensen GJ, Jacobs-Wagner C. 2008. A self-associating protein critical for chromosome attachment, division, and polar organization in caulobacter. Cell 134:956–968. doi:10.1016/j.cell.2008.07.016. 18805089 PMC2614312

[20] Mera PE, Kalogeraki VS, Shapiro L. 2014. Replication initiator DnaA binds at the Caulobacter centromere and enables chromosome segregation. Proc Natl Acad Sci USA 111:16100–16105. doi:10.1073/pnas.1418989111. 25349407 PMC4234595

[21] Lasker K, von Diezmann L, Zhou X, Ahrens DG, Mann TH, Moerner WE, Shapiro L. 2020. Selective sequestration of signalling proteins in a membraneless organelle reinforces the spatial regulation of asymmetry in Caulobacter crescentus. Nat Microbiol 5:418–429. doi:10.1038/s41564-019-0647-7. 31959967 PMC7549192

[22] Berge M, Campagne S, Mignolet J, Holden S, Theraulaz L, Manley S, Allain FH, Viollier PH. 2016. Modularity and determinants of a (bi-)polarization control system from free-living and obligate intracellular bacteria. Elife 5:e20640. doi:10.7554/eLife.20640. 28008852 PMC5182065

[23] Ptacin JL, Gahlmann A, Bowman GR, Perez AM, von Diezmann AR, Eckart MR, Moerner WE, Shapiro L. 2014. Bacterial scaffold directs pole-specific centromere segregation. Proc Natl Acad Sci USA 111:E2046–E2055. doi:10.1073/pnas.1405188111. 24778223 PMC4024888

[24] Zhao WDS, Kowallis KA, Tomares DT, Petitjean HN, Childers WS. 2019. A circuit of protein-protein regulatory interactions enables cell polarity establishment and remodeling. BioRxiv. doi:10.1101/503250.

[25] Viollier PH, Sternheim N, Shapiro L. 2002. Identification of a localization factor for the polar positioning of bacterial structural and regulatory proteins. Proc Natl Acad Sci USA 99:13831–13836. doi:10.1073/pnas.182411999. 12370432 PMC129783

[26] Lawler ML, Larson DE, Hinz AJ, Klein D, Brun YV. 2006. Dissection of functional domains of the polar localization factor PodJ in Caulobacter crescentus. Mol Microbiol 59:301–316. doi:10.1111/j.1365-2958.2005.04935.x. 16359336

[27] Zhang C, Zhao W, Duvall SW, Kowallis KA, Childers WS. 2022. Regulation of the activity of the bacterial histidine kinase PleC by the scaffolding protein PodJ. J Biol Chem 298:101683. doi:10.1016/j.jbc.2022.101683. 35124010 PMC8980812

[28] Hinz AJ, Larson DE, Smith CS, Brun YV. 2003. The Caulobacter crescentus polar organelle development protein PodJ is differentially localized and is required for polar targeting of the PleC development regulator. Mol Microbiol 47:929–941. doi:10.1046/j.1365-2958.2003.03349.x. 12581350

[29] Duerig A, Abel S, Folcher M, Nicollier M, Schwede T, Amiot N, Giese B, Jenal U. 2009. Second messenger-mediated spatiotemporal control of protein degradation regulates bacterial cell cycle progression. Genes Dev 23:93–104. doi:10.1101/gad.502409. 19136627 PMC2632171

[30] Wang J, Moerner WE, Shapiro L. 2021. A localized adaptor protein performs distinct functions at the Caulobacter cell poles. Proc Natl Acad Sci USA 118:e2024705118. doi:10.1073/pnas.2024705118. 33753507 PMC8020655

[31] Balleza E, Kim JM, Cluzel P. 2018. Systematic characterization of maturation time of fluorescent proteins in living cells. Nat Methods 15:47–51. doi:10.1038/nmeth.4509. 29320486 PMC5765880

[32] Schrader JM, Li GW, Childers WS, Perez AM, Weissman JS, Shapiro L, McAdams HH. 2016. Dynamic translation regulation in Caulobacter cell cycle control. Proc Natl Acad Sci USA 113:E6859–E6867. doi:10.1073/pnas.1614795113. 27791168 PMC5098616

[33] Lam H, Schofield WB, Jacobs-Wagner C. 2006. A landmark protein essential for establishing and perpetuating the polarity of a bacterial cell. Cell 124:1011–1023. doi:10.1016/j.cell.2005.12.040. 16530047

[34] Mignolet J, Holden S, Berge M, Panis G, Eroglu E, Theraulaz L, Manley S, Viollier PH. 2016. Functional dichotomy and distinct nanoscale assemblies of a cell cycle-controlled bipolar zinc-finger regulator. Elife 5:e18647. doi:10.7554/eLife.18647. 28008851 PMC5182063

[35] Schofield WB, Lim HC, Jacobs-Wagner C. 2010. Cell cycle coordination and regulation of bacterial chromosome segregation dynamics by polarly localized proteins. EMBO J 29:3068–3081. doi:10.1038/emboj.2010.207. 20802464 PMC2944072

[36] Huitema E, Pritchard S, Matteson D, Radhakrishnan SK, Viollier PH. 2006. Bacterial birth scar proteins mark future flagellum assembly site. Cell 124:1025–1037. doi:10.1016/j.cell.2006.01.019. 16530048

[37] Bowman GR, Comolli LR, Gaietta GM, Fero M, Hong SH, Jones Y, Lee JH, Downing KH, Ellisman MH, McAdams HH, Shapiro L. 2010. Caulobacter PopZ forms a polar subdomain dictating sequential changes in pole composition and function. Mol Microbiol 76:173–189. doi:10.1111/j.1365-2958.2010.07088.x. 20149103 PMC2935252

[38] Anderson-Furgeson JC, Zupan JR, Grangeon R, Zambryski PC. 2016. Loss of PodJ in Agrobacterium tumefaciens leads to ectopic polar growth, branching, and reduced cell division. J Bacteriol 198:1883–1891. doi:10.1128/JB.00198-16. 27137498 PMC4907119

[39] Grangeon R, Zupan J, Jeon Y, Zambryski PC. 2017. Loss of PopZ at activity in agrobacterium tumefaciens by deletion or depletion leads to multiple growth poles, minicells, and growth defects. mBio 8:e01881-17. doi:10.1128/mBio.01881-17. 29138309 PMC5686542

[40] Grangeon R, Zupan JR, Anderson-Furgeson J, Zambryski PC. 2015. PopZ identifies the new pole, and PodJ identifies the old pole during polar growth in Agrobacterium tumefaciens. Proc Natl Acad Sci USA 112:11666–11671. doi:10.1073/pnas.1515544112. 26324921 PMC4577194

[41] Ehrle HM, Guidry JT, Iacovetto R, Salisbury AK, Sandidge DJ, Bowman GR. 2017. Polar organizing protein PopZ is required for chromosome segregation in Agrobacterium tumefaciens. J Bacteriol 199:e00111-17. doi:10.1128/JB.00111-17. 28630129 PMC5553026

[42] Howell M, Aliashkevich A, Salisbury AK, Cava F, Bowman GR, Brown PJB. 2017. Absence of the polar organizing protein PopZ results in reduced and asymmetric cell division in Agrobacterium tumefaciens. J Bacteriol 199:e00101-17. doi:10.1128/JB.00101-17. 28630123 PMC5553032

[43] Pfeiffer D, Toro-Nahuelpan M, Bramkamp M, Plitzko JM, Schuler D. 2019. The polar organizing protein PopZ is fundamental for proper cell division and segregation of cellular content in Magnetospirillum gryphiswaldense. mBio 10:e02716-18. doi:10.1128/mBio.02716-18. 30862753 PMC6414705

[44] Ettema TJ, Andersson SG. 2009. The alpha-proteobacteria: the Darwin finches of the bacterial world. Biol Lett 5:429–432. doi:10.1098/rsbl.2008.0793. 19324639 PMC2679921

[45] Zhang H, Ji X, Li P, Liu C, Lou J, Wang Z, Wen W, Xiao Y, Zhang M, Zhu X. 2020. Liquid-liquid phase separation in biology: mechanisms, physiological functions and human diseases. Sci China Life Sci 63:953–985. doi:10.1007/s11427-020-1702-x. 32548680

[46] Saurabh S, Chong TN, Bayas C, Dahlberg PD, Cartwright HN, Moerner WE, Shapiro L. 2022. ATP-responsive biomolecular condensates tune bacterial kinase signaling. Sci Adv 8:eabm6570. doi:10.1126/sciadv.abm6570. 35171683 PMC8849385

[47] Tan W, Cheng S, Li Y, Li XY, Lu N, Sun J, Tang G, Yang Y, Cai K, Li X, Ou X, Gao X, Zhao GP, Childers WS, Zhao W. 2022. Phase separation modulates the assembly and dynamics of a polarity-related scaffold-signaling hub. Nat Commun 13:7181. doi:10.1038/s41467-022-35000-2. 36418326 PMC9684454

[48] Thanbichler M, Iniesta AA, Shapiro L. 2007. A comprehensive set of plasmids for vanillate- and xylose-inducible gene expression in Caulobacter crescentus. Nucleic Acids Res 35:e137. doi:10.1093/nar/gkm818. 17959646 PMC2175322

[49] Schindelin J, Arganda-Carreras I, Frise E, Kaynig V, Longair M, Pietzsch T, Preibisch S, Rueden C, Saalfeld S, Schmid B, Tinevez JY, White DJ, Hartenstein V, Eliceiri K, Tomancak P, Cardona A. 2012. Fiji: an open-source platform for biological-image analysis. Nat Methods 9:676–682. doi:10.1038/nmeth.2019. 22743772 PMC3855844

[50] Ducret A, Quardokus EM, Brun YV. 2016. MicrobeJ, a tool for high throughput bacterial cell detection and quantitative analysis. Nat Microbiol 1:16077. doi:10.1038/nmicrobiol.2016.77. 27572972 PMC5010025

[51] Schrader JM, Shapiro L. 2015. Synchronization of Caulobacter crescentus for investigation of the bacterial cell cycle. J Vis Exp 98:52633. doi:10.3791/52633. PMC4541484 25938623

[52] Joshi KK, Battle CM, Chien P. 2018. Polar localization hub protein PopZ restrains adaptor-dependent ClpXP proteolysis in Caulobacter crescentus. J Bacteriol 200:e00221-18. doi:10.1128/JB.00221-18. 30082457 PMC6153658

[53] Childers WS, Xu Q, Mann TH, Mathews II, Blair JA, Deacon AM, Shapiro L. 2014. Cell fate regulation governed by a repurposed bacterial histidine kinase. PLoS Biol 12:e1001979. doi:10.1371/journal.pbio.1001979. 25349992 PMC4211667

[54] Guzzo M, Castro LK, Reisch CR, Guo MS, Laub MT. 2020. A crispr interference system for efficient and rapid gene knockdown in Caulobacter crescentus. mBio 11:e02415-19. doi:10.1128/mBio.02415-19. 31937638 PMC6960281

[55] Erdős G, Pajkos M, Dosztányi Z. 2021. IUPred3: prediction of protein disorder enhanced with unambiguous experimental annotation and visualization of evolutionary conservation. Nucleic Acids Res 49:W297–W303. doi:10.1093/nar/gkab408. 34048569 PMC8262696

[56] Marioni JC, Mason CE, Mane SM, Stephens M, Gilad Y. 2008. RNA-seq: an assessment of technical reproducibility and comparison with gene expression arrays. Genome Res 18:1509–1517. doi:10.1101/gr.079558.108. 18550803 PMC2527709

[57] Langmead B, Salzberg SL. 2012. Fast gapped-read alignment with Bowtie 2. Nat Methods 9:357–359. doi:10.1038/nmeth.1923. 22388286 PMC3322381

[58] Wang L, Feng Z, Wang X, Wang X, Zhang X. 2010. DEGseq: an R package for identifying differentially expressed genes from RNA-seq data. Bioinformatics 26:136–138. doi:10.1093/bioinformatics/btp612. 19855105

[59] Kumar S, Stecher G, Li M, Knyaz C, Tamura K. 2018. MEGA X: Molecular Evolutionary Genetics Analysis across computing platforms. Mol Biol Evol 35:1547–1549. doi:10.1093/molbev/msy096. 29722887 PMC5967553

[60] Ludwiczak J, Winski A, Szczepaniak K, Alva V, Dunin-Horkawicz S. 2019. DeepCoil-a fast and accurate prediction of coiled-coil domains in protein sequences. Bioinformatics 35:2790–2795. doi:10.1093/bioinformatics/bty1062. 30601942

