# Supplementary material for: Scaffold-Scaffold Interaction Facilitates Cell Polarity Development in Caulobacter crescentus
Source: mBio. 2023 Mar 27;14(2):e03218-22. doi: 10.1128/mbio.03218-22 (PMC10127582; doi:10.1128/mbio.03218-22)
Supplement: FIG S1 [file mbio.03218-22-s0001.pdf]

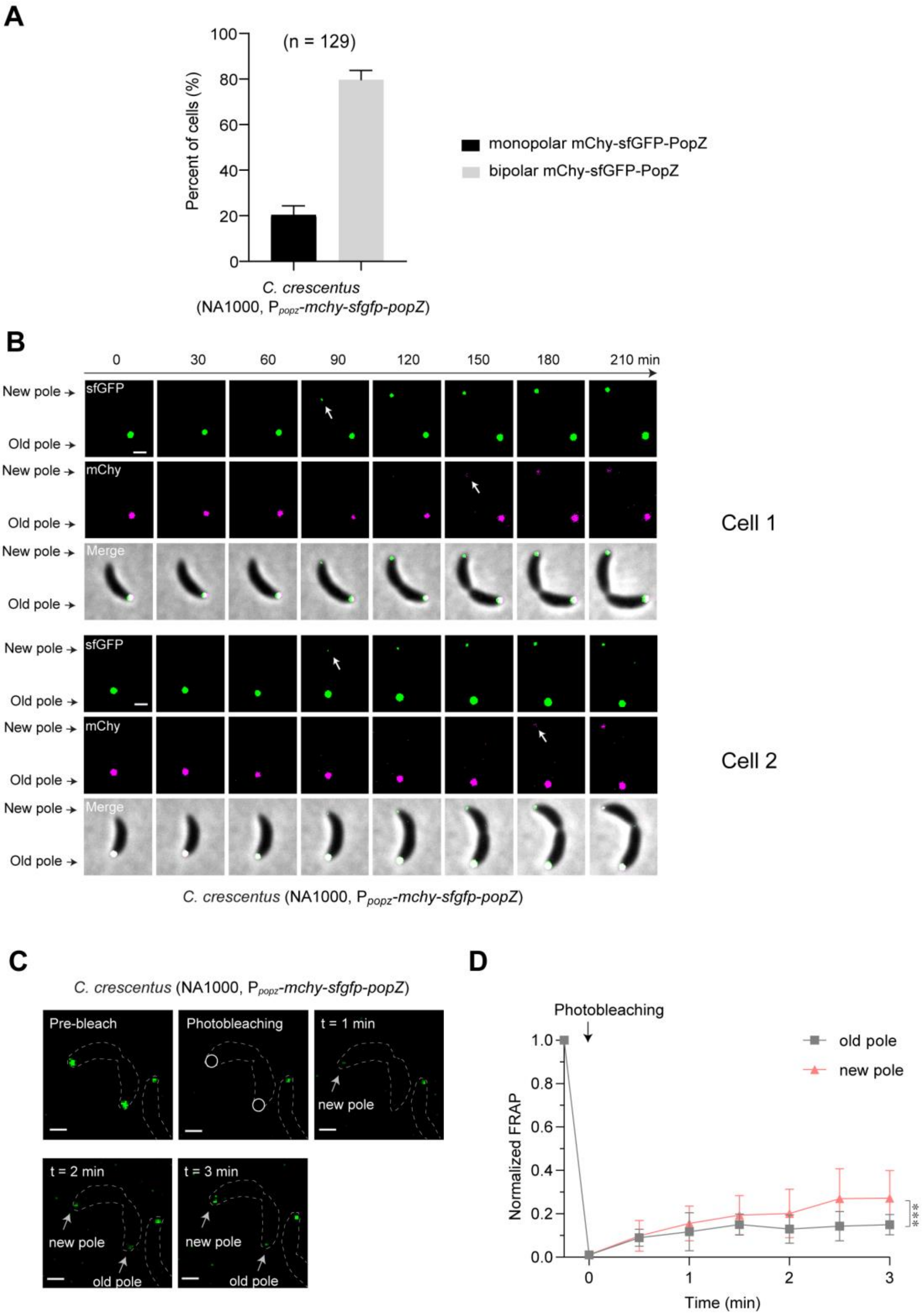

Supplementary Figure 1. Pole-specific FRAP analysis suggests that the newly synthesized PopZ could be accumulated at both cell poles. A, Expression of mCherry-sfGFP-PopZ shows a bipolar localization pattern as that of the untagged

PopZ (1) in predivisional *C. crescentus* cells. In the pre-divisional cell stage after synchronization, the subcellular localization of mCherry-sfGFP-PopZ was monitored in LN001 (NA1000, P_{popZ}-*mcherry-sfgfp-popZ*) strain. The fluorescence tagged *popZ* gene was integrated in the chromosome of *C. crescentus* under the control of the native promoter. **B**, PopZ accumulates at the new pole through *de-novo* synthesis. Two more cells are shown here as in Figure 1C. **C**, FRAP analysis reveals that the fluorescence intensities of mCherry-sfGFP-PopZ condensates were partially recovered after photobleaching in *C. crescentus*. The expression of mCherry-sfGFP-PopZ was driven by the endogenous *popZ* promoter in the *C. crescentus* chromosome. The cell pole regions with all the PopZ accumulated were selected for photobleaching (indicated by the white circles). One representative bleached cell is shown. White arrows indicate the fluorescence recovery at the new cell pole or old cell pole. **D**, Quantification of the FRAP analyses in panel A. The recovery curve was generated by averaging the signals of 7 cells. A total of 27% and 15% recovery within 3 minutes were shown at the new cell pole and the old cell pole, respectively. The fluorescence intensity of pre-bleached foci was normalized as 100%. Taking the average fluorescent signal of three PopZ foci without bleaching as a reference, each signal intensity of the experimental group was first normalized with the signal intensity of the reference group at each time point. ***, $P < 0.001$ determined by two-way ANOVA. All scale bars, 1 μm.

SUPPLEMENTARY REFERENCES

1. Ebersbach G, Briegel A, Jensen GJ, Jacobs-Wagner C. 2008. A self-associating protein critical for chromosome attachment, division, and polar organization in caulobacter. Cell 134:956-68.
