# Supplementary material for: Scaffold-Scaffold Interaction Facilitates Cell Polarity Development in Caulobacter crescentus
Source: mBio. 2023 Mar 27;14(2):e03218-22. doi: 10.1128/mbio.03218-22 (PMC10127582; doi:10.1128/mbio.03218-22)
Supplement: FIG S2 [file mbio.03218-22-s0002.pdf]

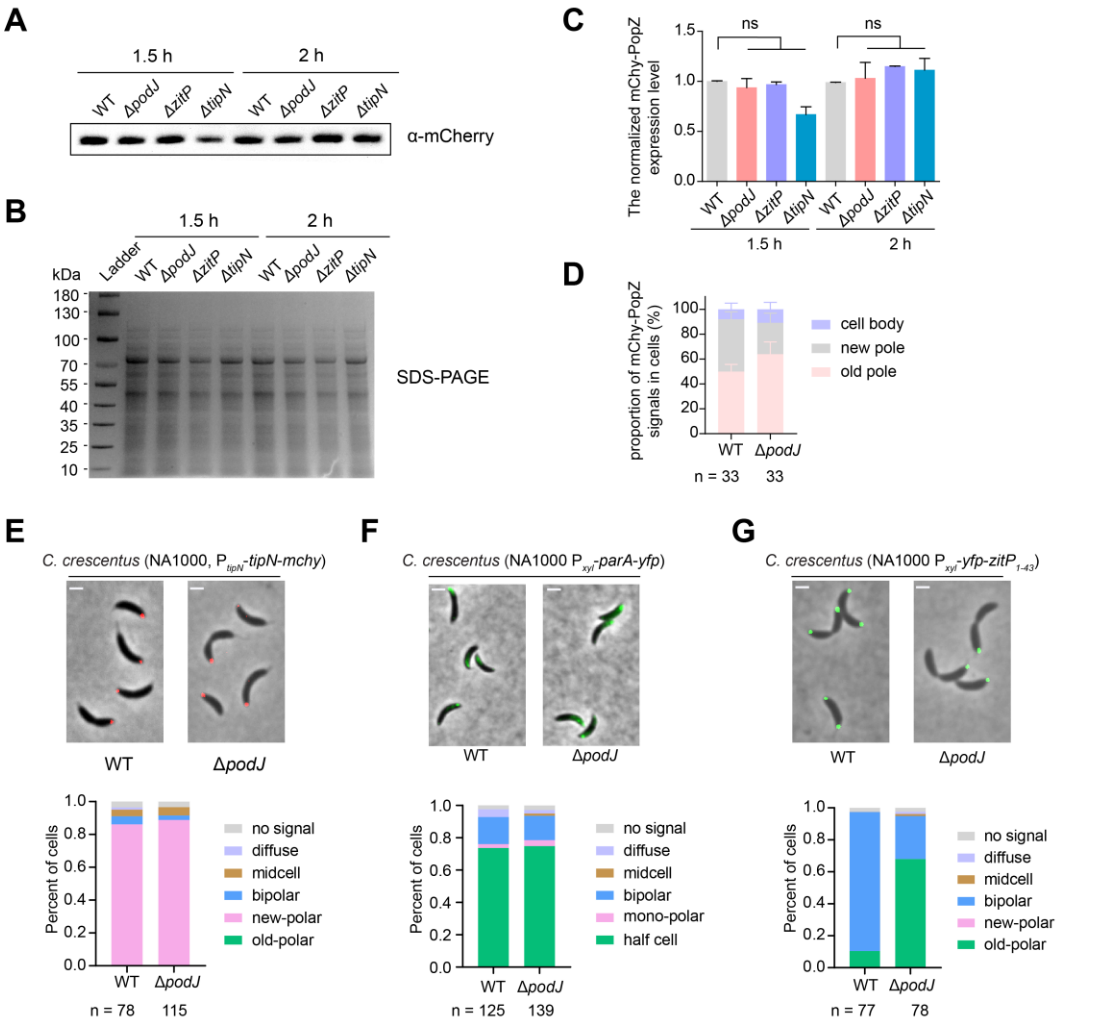

Supplementary Figure 2. The PodJ effect upon PopZ localization is not through the regulation of PopZ expression nor via the indirect regulation of TipN or ParA localization. A, Western blot analysis of PopZ expression in *C. crescentus* mutation strains. The *C. crescentus* cells expressing a sole copy of *mcherry-popZ* were synchronized and transferred to the liquid M2G medium. The same cell mass was taken from different mutation strains at 1.5 h and 2 h, respectively. The expression level of mCherry-PopZ was analyzed by western blotting using an anti-mCherry antibody. **B**, Detection of the total proteins in cells. Total proteins from the samples in panel A were detected by SDS-PAGE. **C**, Quantification in *C. crescentus* mutation strains reveals that the expression of PopZ is not regulated by PodJ, ZitP or TipN. The grayscale values of western blot and SDS-PAGE results were quantified with

FIJI/ImageJ (2). The corresponding expression levels of mCherry-PopZ in *C. crescentus* strains were normalized by western blot values against the total signal of SDS-PAGE lanes, respectively. Two independent experiments were performed and statistical analyses were executed using GraphPad Prism 5.0. Statistically significant differences were determined using Welch's unpaired *t*-test. ns, $P \geq 0.05$. **D**, Quantitative analysis of mCherry-PopZ signal at the old pole, cell body and the new pole in Δ*podJ* and the wild-type cells. Each experiment was performed in three biological replicates. A total of 33 cells (n) were calculated for each sample set. Corresponding to Figure 2A. **E** and **F**, The subcellular localization of TipN and ParA is not modulated by PodJ. The *tipN* and *parA* gene were integrated into the chromosome, respectively, to observe their subcellular localization changes after deletion of *podJ* in *C. crescentus*. The fluorescent tags were integrated at the 3' end of the *tipN* and *parA* gene, respectively. Quantification of protein localization patterns are shown on the below. **G**, The subcellular localization of YFP-ZitP$_{1\text{-}43}$ may be regulated by PopZ instead of PodJ. Fluorescently labeled *zitP*$_{1\text{-}43}$ (5' end labeling) was used as described in (3). The majority of YFP-ZitP$_{1\text{-}43}$ localization was changed from bipolar to old-polar after deletion of *podJ*, which is likely the result of the loss of bipolar PopZ, rather than the cause (3). All scale bars, 1 µm.

SUPPLEMENTARY REFERENCES

2. Schindelin J, Arganda-Carreras I, Frise E, Kaynig V, Longair M, Pietzsch T, Preibisch S, Rueden C, Saalfeld S, Schmid B, Tinevez JY, White DJ, Hartenstein V, Eliceiri K, Tomancak P, Cardona A. 2012. Fiji: an open-source platform for biological-image analysis. Nat Methods 9:676-82.
3. Berge M, Campagne S, Mignolet J, Holden S, Theraulaz L, Manley S, Allain FH, Viollier PH. 2016. Modularity and determinants of a (bi-)polarization control system from free-living and obligate intracellular bacteria. Elife 5.
