# Supplementary material for: Scaffold-Scaffold Interaction Facilitates Cell Polarity Development in Caulobacter crescentus
Source: mBio. 2023 Mar 27;14(2):e03218-22. doi: 10.1128/mbio.03218-22 (PMC10127582; doi:10.1128/mbio.03218-22)
Supplement: FIG S3 [file mbio.03218-22-s0003.pdf]

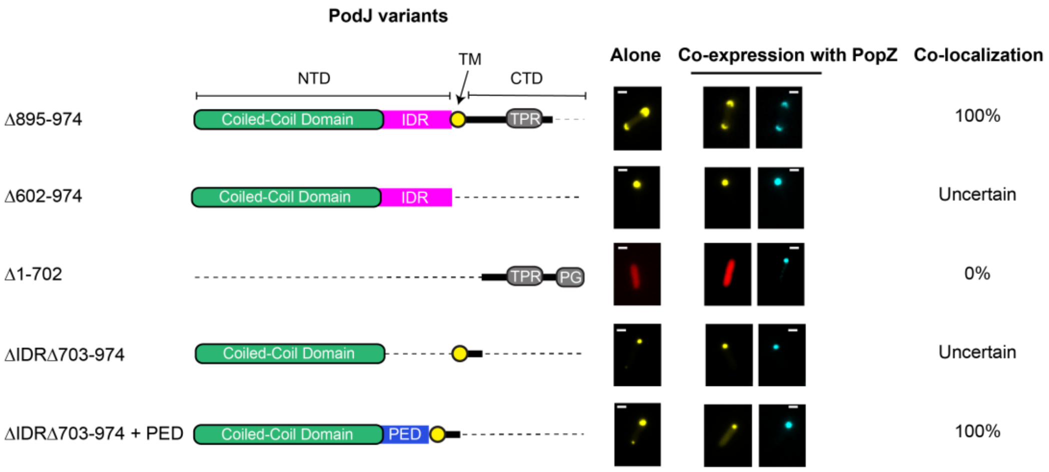

Supplementary Figure 3. Identification of the N-terminus of PodJ as the possible interaction region with PopZ. The schematic diagrams of truncated PodJ proteins are shown on the left panel. The cells with co-localized PopZ and PodJ variants are calculated and shown on the right panel. At least 200 cells were counted in each sample. TM, transmembrane domain; IDR, intrinsically disordered region; TPR, tandem tetratricopeptide repeats; PG, peptidoglycan binding domain; PED, intrinsically disordered region in PopZ ($PopZ_{24\text{-}102}$) (4). All scale bars, 1 µm.

SUPPLEMENTARY REFERENCES

4. Holmes JA, Follett SE, Wang H, Meadows CP, Varga K, Bowman GR. 2016. *Caulobacter* PopZ forms an intrinsically disordered hub in organizing bacterial cell poles. Proc Natl Acad Sci U S A 113:12490-12495.
