# Supplementary material for: Scaffold-Scaffold Interaction Facilitates Cell Polarity Development in Caulobacter crescentus
Source: mBio. 2023 Mar 27;14(2):e03218-22. doi: 10.1128/mbio.03218-22 (PMC10127582; doi:10.1128/mbio.03218-22)
Supplement: FIG S4 [file mbio.03218-22-s0004.pdf]

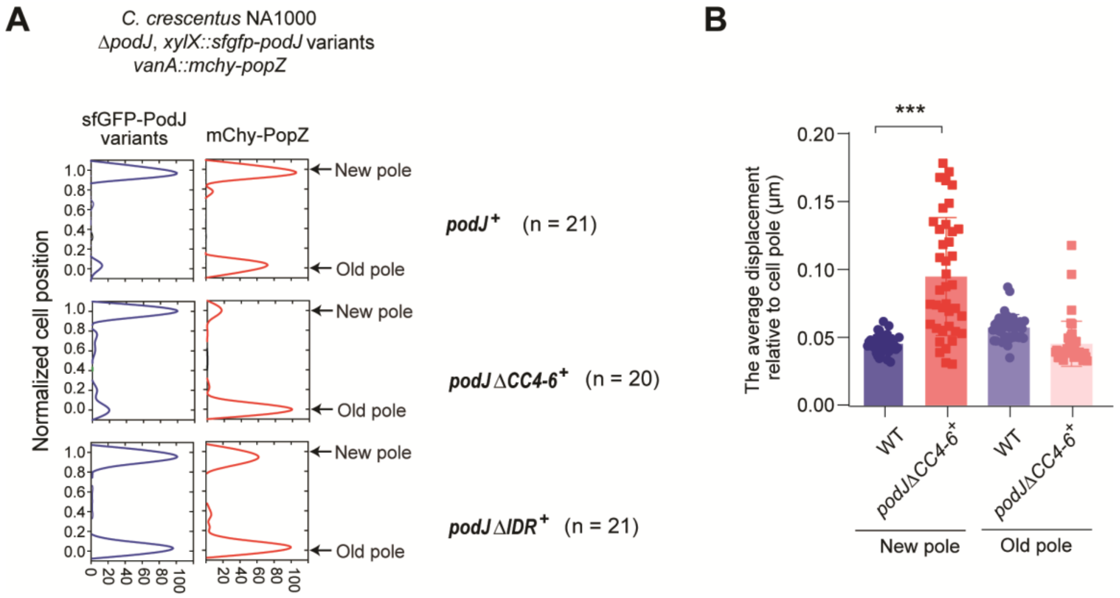

Supplementary Figure 4. The PodJ variant that lack of CC4-6 is able to accumulate but causes a higher mobility of CFP-ParB at the new cell pole. **A**, The PodJ variant is able to accumulate at the new cell pole whereas PopZ is unable to accumulate at the new cell pole when CC4-6 of PodJ is lacking. The sfGFP-PodJ variants and mCherry-PopZ were expressed as the sole copy in *C. crescentus* chromosome under the P_{xyl} or P_{van} promoter. Relatively low concentration of inducer (0.003% xylose or 0.05 mM vanillate) was used for 3 hours in this assay. Quantitative analyses were performed for the signal intensities of PodJ variants and PopZ along the cell lengths. At least 20 cells (n) were calculated for each test set. Data are normalized with the highest intensity as 100% in cells. **B**, The CFP-ParB focus has a higher mobility at the new pole of *C. crescentus* with PodJ variant that lack of CC4-6. The CFP-ParB, mCherry-PopZ, and PodJΔCC4-6 were expressed as the sole copy under the endogenous promoter, respectively, in wild-type *C. crescentus* JP468 (NA1000, P_{parB}-*cfp-parB*, P_{popZ}-*mcherry-popZ*) or in LN104 (NA1000 Δ*podJ*, P_{parB}-*cfp-parB*, P_{popZ}-*mcherry-popZ*, P_{podJ}-*podJΔCC4-6*). Analysis of the average displacements of CFP-ParB foci to the cell poles is shown. Each point refers to an average value of displacements for a focus during 22 minutes with an interval of 2 minutes. At least 40

CFP-ParB foci were calculated for each sample. Statistically significant differences were determined using Welch's unpaired *t*-test. ***, $P < 0.001$.
