# Supplementary material for: Scaffold-Scaffold Interaction Facilitates Cell Polarity Development in Caulobacter crescentus
Source: mBio. 2023 Mar 27;14(2):e03218-22. doi: 10.1128/mbio.03218-22 (PMC10127582; doi:10.1128/mbio.03218-22)
Supplement: FIG S5 [file mbio.03218-22-s0005.pdf]

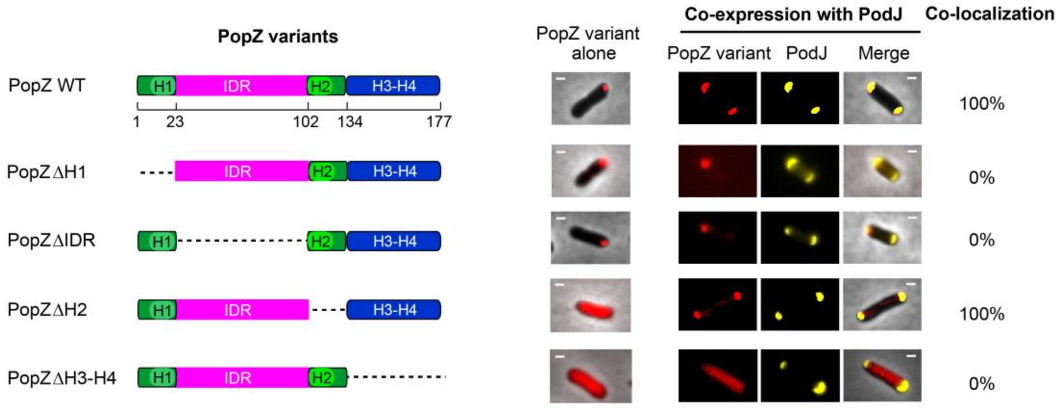

Supplementary Figure 5. Identification of the domains of PopZ interaction with PodJ by heterologous co-expression experiments. The PopZ variants were expressed alone or co-expressed with PodJ in E. coli to observe their changes of subcellular localization. Co-localization results showed that PopZ_H2 domain is not responsible for the interaction with PodJ. Scale bars, 1 µm.
