# Supplementary material for: Scaffold-Scaffold Interaction Facilitates Cell Polarity Development in Caulobacter crescentus
Source: mBio. 2023 Mar 27;14(2):e03218-22. doi: 10.1128/mbio.03218-22 (PMC10127582; doi:10.1128/mbio.03218-22)
Supplement: FIG S6 [file mbio.03218-22-s0006.pdf]

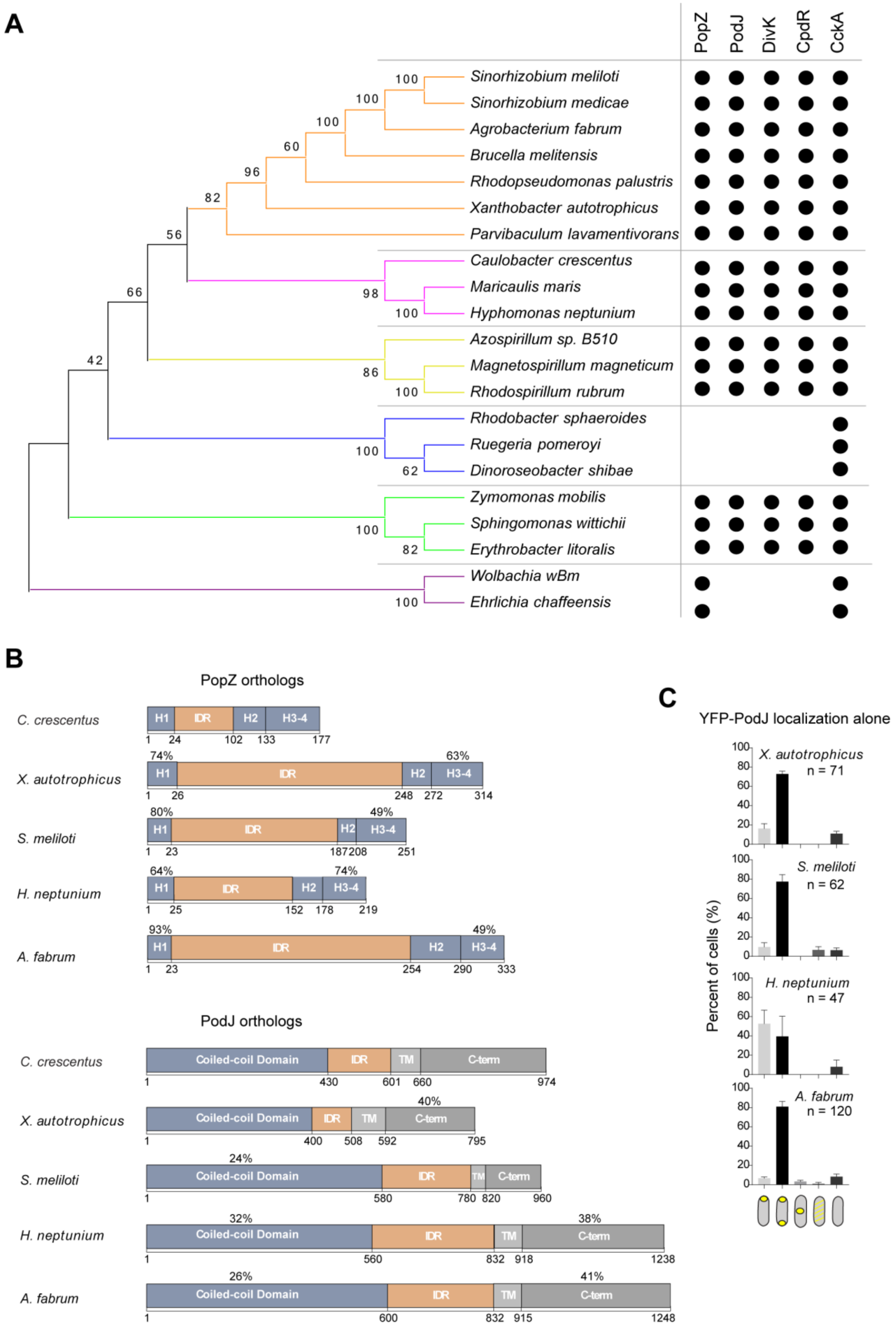
A
PopZ
PodJ
DivK
CpdR
CckA
Sinorhizobium meliloti
Sinorhizobium medicae
Agrobacterium fabrum
Brucella melitensis
Rhodopseudomonas palustris
Xanthobacter autotrophicus
Parvibaculum lavamentivorans
Caulobacter crescentus
Maricaulis maris
Hyphomonas neptunium
Azospirillum sp. B510
Magnetospirillum magneticum
Rhodospirillum rubrum
Rhodobacter sphaeroides
Ruegeria pomeroyi
Dinoroseobacter shibae
Zymomonas mobilis
Sphingomonas wittichii
Erythrobacter litoralis
Wolbachia wBm
Ehrlichia chaffeensis
B
PopZ orthologs
C. crescentus
X. autotrophicus
S. meliloti
H. neptunium
A. fabrum
H1
IDR
H2
H3-4
PodJ orthologs
Coiled-coil Domain
TM
C-term
C
YFP-PodJ localization alone
Percent of cells (%)
X. autotrophicus n = 71
S. meliloti n = 62
H. neptunium n = 47
A. fabrum n = 120

Supplementary Figure 6. Systematic analyses of PodJ and PopZ orthologs in α-proteobacteria. A, Protein orthologous analyses reveals that PopZ and PodJ are encoded in a subset of α-proteobacteria. The corresponding orthologs in α-proteobacteria were obtained through BLASTP program using *C. crescentus* proteins as the query sequences, respectively (identity > 20%, coverage > 30%). Phylogenetic tree was built by MEGA X (5) using 21 amino acid sequences of CckA with Neighbor-Joining method (bootstrap: 1,000). Black dots indicate the presence of corresponding orthologs. **B**, Domain analyses of PopZ and PodJ orthologs in selected α-proteobacteria. Domains of PopZ and PodJ orthologs were annotated by alignments of protein sequences. Protein identities that higher than 20% (compared with *C. crescentus* PopZ or PodJ ortholog) were marked above the schematic domains. Prediction of coiled-coil regions in PopZ and PodJ orthologs was achieved using DeepCoil2 (6). Prediction of intrinsically disordered regions (IDR) was achieved using IUPred3 (7). TM, transmembrane domain. **C**, Quantitative analyses of YFP-PodJ localization patterns in *E. coli*. At least 47 cells (n) were calculated in each sample. The corresponding PodJ in α-proteobacteria were obtained through BLASTP program using *C. crescentus* PodJ as the query sequences on KEGG database. *C. crescentus* PodJ, CCNA_02125; *X. autotrophicus* PodJ, Xaut_3064; *S. meliloti* PodJ, SMc02230; *H. neptunium* PodJ, HNE_0666; *A. fabrum* PodJ, Atu0499.

SUPPLEMENTARY REFERENCES

5. Kumar S, Stecher G, Li M, Knyaz C, Tamura K. 2018. MEGA X: Molecular Evolutionary Genetics Analysis across Computing Platforms. Mol Biol Evol 35:1547-1549.
6. Ludwiczak J, Winski A, Szczepaniak K, Alva V, Dunin-Horkawicz S. 2019. DeepCoil-a fast and accurate prediction of coiled-coil domains in protein sequences. Bioinformatics 35:2790-2795.
7. Erdos G, Pajkos M, Dosztanyi Z. 2021. IUPred3: prediction of protein disorder enhanced with unambiguous experimental annotation and visualization of

evolutionary conservation. Nucleic Acids Res 49:W297-W303.
