# Supplementary material for: Scaffold-Scaffold Interaction Facilitates Cell Polarity Development in Caulobacter crescentus
Source: mBio. 2023 Mar 27;14(2):e03218-22. doi: 10.1128/mbio.03218-22 (PMC10127582; doi:10.1128/mbio.03218-22)
Supplement: FIG S7 [file mbio.03218-22-s0007.pdf]

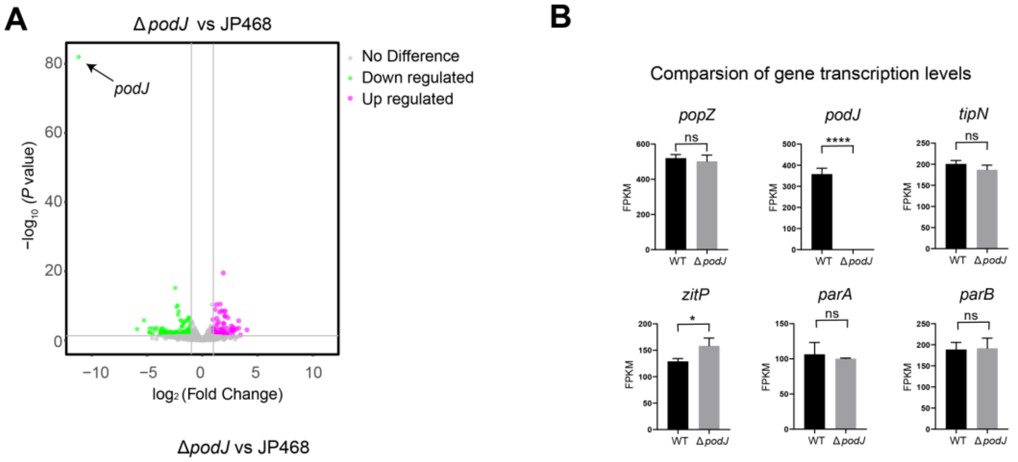

ΔpodJ vs JP468

Quantified genes	Total differentially expressed genes	Up-regulated	Down-regulated
4076	316	135	181

Supplementary Figure 7. Transcriptome analysis after deletion of *podJ* gene.

A, Volcano plot of the differentially expressed genes between Δ*podJ* and JP468 strains. A total of 6 samples with 3 biological repeats were used for RNA extraction and transcriptome sequencing in 1.5 h after synchronization. The changes of gene transcriptions were displayed after deletion of *podJ* in JP468 strain. Magenta dots represent the significantly up-regulated genes, green dots represent the significantly down-regulated genes, and grey dots indicate the non-significance of difference in gene transcription between the two strains. The numbers of differentially regulated genes in Δ*podJ* strain were summarized as below. For more details please see Table S2. **B**, The transcription levels of *popZ*, *tipN*, *parA*, and *parB* are not affected by the deletion of *podJ*. The results indicate that the promotion of bipolar PopZ by PodJ was not through the regulation of PopZ expression, nor via the indirectly regulation of expression of TipN or ParA. The transcription level of *zitP* is even slightly increased in Δ*podJ*, indicating the promotion of bipolar PopZ by PodJ was not through the indirectly regulation of ZitP expression either. FPKM: Fragments Per Kilobase of

exon model per Million mapped fragments. ****, $P < 0.0001$; **, $P < 0.01$; *, $P < 0.05$; ns, $P \geq 0.05$ by two-tailed paired *t*-test.
