# Supplementary material for: Scaffold-Scaffold Interaction Facilitates Cell Polarity Development in Caulobacter crescentus
Source: mBio. 2023 Mar 27;14(2):e03218-22. doi: 10.1128/mbio.03218-22 (PMC10127582; doi:10.1128/mbio.03218-22)
Supplement: TABLE S3 [file mbio.03218-22-s0010.pdf]

SUPPLEMENTARY TABLES

Supplementary Table 3A. Plasmids used in this study.

**Plasmid**	**Plasmid information**[a]	**Source or reference**
pLN053	Kan^R; pXYFPN-2-P*PopZ*-mCherry-sfGFP-PopZ	This study
pLN048	Kan^R; pXYFPN-2-RpoC-mCherry-sfGFP	This study
pLN051	Kan^R; pXYFPN-2-P*PodJ*-PodJ	This study
pLN055	Kan^R; pXYFPN-2-P*PodJ*-PodJΔCC4-6	This study
pLN058	Kan^R; pXYFPN-2-P*PodJ*-PodJΔIDR	This study
pLNS001	Kan^R; pBXMCS2-sgRNA-ΔPodJ-UA1-DA1-*sp*Cas9M	This study
pLNS002	Kan^R; pBXMCS2-sgRNA-$\Delta ZitP_{6\text{-}283}$-UA2-DA2-*sp*Cas9M	This study
pLNS003	Kan^R; pBXMCS2-sgRNA-$\Delta TipN_{18\text{-}825}$-UA3-DA3-*sp*Cas9M	This study
pLNS004	Kan^R; pBXMCS2-sgRNA-ΔPodJ-UA1-DA1	This study
pLNS005	Kan^R; pBXMCS2-sgRNA-$\Delta ZitP_{6\text{-}283}$-UA1-DA1-*sp*Cas9M	This study
pLNS006	Kan^R; pBXMCS2-sgRNA-$\Delta TipN_{18\text{-}825}$-UA1-DA1-*sp*Cas9M	This study
AP139	Amp^R; pBAD-mCherry-PopZ	(8)
pLN061	Amp^R; pBAD-mCherry-PopZ (*A. fabrum*)	This study
pLN063	Amp^R; pBAD-mCherry-PopZ (*S. meliloti*)	This study
pLN065	Amp^R; pBAD-mCherry-PopZ (*H. neptunium*)	This study
pLN067	Amp^R; pBAD-mCherry-PopZ (*X. autotrophicus*)	This study
pWZ012	$Spec^R$; pCDF-YFP-PodJ	(8)
pLN056	$Spec^R$; pCDF-YFP-TipN	This study
pLN057	$Spec^R$; pCDF-YFP-ZitP	This study
pLN062	$Spec^R$; pCDF-YFP-PodJ (*A. fabrum*)	This study
pLN064	$Spec^R$; pCDF-YFP-PodJ (*S. meliloti*)	This study
pLN066	$Spec^R$; pCDF-YFP-PodJ (*H. neptunium*)	This study
pLN068	$Spec^R$; pCDF-YFP-PodJ (*X. autotrophicus*)	This study
pLN080	$Spec^R$; pCDF-YFP-PodJΔ703-974	This study
pLN081	$Spec^R$; pCDF-YFP-PodJΔCC4-6	This study
pLN082	$Spec^R$; pCDF-YFP-$PodJ_{CC4\text{-}6}$	This study
pLN083	$Spec^R$; pCDF-YFP-$PodJ_{IDR}$	This study
pLN084	$Spec^R$; pCDF-YFP-PodJΔ895-974	This study
pLN085	$Spec^R$; pCDF-YFP-PodJΔ602-974	This study
pLN086	$Spec^R$; pCDF-YFP-PodJΔ1-702	This study
pWZ096-cys	Amp^R; pTEV5-Cys-$PodJ_{471\text{-}635}$	(9)
pWZ098-cys	Amp^R; pTEV5-Cys-$PodJ_{250\text{-}430}$	(9)

pWT187	Kan^R; pET28a-mCherry-PopZ	(8)
pLN087	Kan^R; pET28a-PopZ	This study
pLN088	$Spec^R$; pCDF-YFP-PodJΔIDRΔ703-974	This study
pLN089	$Spec^R$; pCDF-YFP-PodJΔIDRΔ703-974+PED ($PopZ_{24-102}$)	This study
pWT243	Chl^R; pVCHYN-6-P_{van}-mCherry-SpmX	(8)
pLN090	Chl^R; pVCHYN-6-P_{van}-mCherry-sfGFP-PopZ	This study
pWT147	Kan^R; pXYFPN-2-P_{xyl}-sfGFP-PodJ_N	(8)
pWT205	Kan^R; pBXMCS2-P_{xyl}-YFP-PodJ	(8)
pLN091	Kan^R; pXYFPN-2-P_{xyl}-YFP-$ZitP_{1-43}$	This study
pLN092	Kan^R; pXYFPN-2-P_{tipN}-TipN-mCherry	This study
pLN094	Kan^R; pXYFPN-2-P_{xyl}-ParA-YFP	This study
pWZ0113	Kan^R; pXYFPN-2-P_{xyl}-sfGFP-PodJ	(8)

[a]Abbreviations: Kan, kanamycin; Chl, chloramphenicol; Spec, spectinomycin; Amp, ampicillin; R, resistance.

Supplementary Table 3B. Bacterial strains used in this study.

**Strains**	**Description**	**Construction, source or reference**
NA1000	*C. crescentus* wild-type strain	Lucy Shapiro lab
JP468	NA1000, P_{parB}-*cfp-parB*, P_{popZ}-*mcherry-popZ*	(10)
LN001	NA1000, P_{popZ}-*mcherry-sfgfp-popZ*	Single integration of pLN053 into NA1000
LN002	NA1000, P_{rpoC}-*rpoC-mcherry-sfgfp*	Single integration of pLN048 into NA1000
LN100	NA1000 Δ*podJ*	One step knockout of *podJ* by pLNS001 in NA1000
LN101	NA1000 Δ*podJ*, P_{parB}-*cfp-parB*, P_{popZ}-*mcherry-popZ*	One step knockout of *podJ* by pLNS001 in JP468
LN102	NA1000 Δ*podJ,* P_{parB}-*cfp-parB*, P_{popZ}-*mcherry-popZ*, P_{podJ}-*podJ*	Single integration of pLN051 into LN101
LN103	NA1000 Δ*podJ,* P_{parB}-*cfp-parB*, P_{popZ}-*mcherry-popZ*, P_{podJ}-*podJΔIDR*	Single integration of pLN058 into LN101
LN104	NA1000 Δ*podJ,* P_{parB}-*cfp-parB*, P_{popZ}-*mcherry-popZ*, P_{podJ}-*podJΔCC4-6*	Single integration of pLN055 into LN101
LN105	NA1000 Δ$zitP_{6\text{-}283}$, P_{parB}-*cfp-parB*, P_{popZ}-*mcherry-popZ*	One step knockout of $zitP_{6\text{-}283}$ by pLNS002 in JP468
LN106	NA1000 Δ$tipN_{18\text{-}825}$, P_{parB}-*cfp-parB*, P_{popZ}-*mcherry-popZ*	One step knockout of $tipN_{18\text{-}825}$ by pLNS003 in JP468
LN107	NA1000, P_{xyl}-*yfp*-$zitP_{1\text{-}43}$	Single integration of pLN091 into NA1000
LN108	NA1000 Δ*podJ*, P_{xyl}-*yfp*-$zitP_{1\text{-}43}$	Single integration of pLN091 into LN100
LN109	NA1000, P_{tipN}-*tipN-mcherry*	Single integration of pLN092 into NA1000
LN111	NA1000 Δ*podJ*, P_{tipN}-*tipN-mcherry*	Single integration of pLN092 into LN100
LN114	NA1000, P_{xyl}-*parA-yfp*	Single integration of pLN094 into NA1000
LN115	NA1000 Δ*podJ*, P_{xyl}-*parA-yfp*	Single integration of pLN094 into LN100
DH5α	Bacterial cloning strain	Novagen
BL21(DE3)	Bacterial expression strain	Novagen

Supplementary Table 3C. Oligonucleotides used in this study.

Primer	Sequence	Plasmids
NLP-001	atataagcttagaccaagggcgagtacatcatcgacg	pLN048
NLP-002	gctaccactgccaccttcggcgtccgaaagcgcg	
NLP-003	ccgaaggtggcagtggtagcatggtgagcaagggcgaggagg	
NLP-004	cgggcgcctatttgtacagttcatccataccatgcgtgatgcc	
NLP-005	actgtacaaataggcgcccgaaacagtcgaagac	
NLP-006	attaattaaggcaatgcgcaatgacatcgtttgcatactccc	
NLP-007	tcattgcgcattgccttaattaatatgcatggtaccttaagatctcgagctccg	
NLP-008	actcgcccttggtctaagcttatataaaaactgttgtaattcattaagcattctgccga	
NLP-009	aacagtttttaccgtcgcaacgtttgcgtgaaaa	pLN053
NLP-010	ttgctcaccatgtgcggggccgtcgtaaagag	
NLP-011	ggccccgcacatggtgagcaagggcgaggag	
NLP-012	ttaattaaggcttaggcgccgcgtccccg	
NLP-013	gcggcgcctaagccttaattaatatgcatggtaccttaagatctcgagct	
NLP-014	cgttgcgacggtaaaaactgttgtaattcattaagcattctgccgacatggaagc	
NLP-015	ataagcttaggtgcggcgccgaccatga	pLN051
NLP-016	ttaattaaggccccttcccggcaaagcgcg	
NLP-017	ccgggaaggggccttaattaatatgcatggtaccttaagatctcgagctcc	
NLP-018	ggcgccgcacctaagcttatataaaaactgttgtaattcattaagcattctgccgacat	
NLP-019	cctggatcagcgccaggaactggtcgaccgca	pLN055
NLP-020	gcgctgatccagggcctgg	
NLP-021	ccagcggcaaggccaagtcggcga	pLN058
NLP-022	ttgccgctggaacgttcgtggcgttg	
NLP-023	gtcggccaccatgaagcctaagaagcgccaaccg	pLN056
NLP-024	attgagatctgcctaggccagatcgccgctcg	
NLP-025	atctggcctaggcagatctcaattggatatcggccggccac	
NLP-026	taggcttcatggtggccgaccggtgct	
NLP-027	gtcggccaccgtggggttcgttcgattcgcgg	pLN057
NLP-028	aattgagatctgctcattcatgatgagcgggttcgtgagagtct	
NLP-029	catcatgaatgagcagatctcaattggatatcggccggc	
NLP-030	cgaaccccacggtggccgaccggtg	
NLP-031	gaccgcgtcgcgcgcccagcggcgc	pLNS004
NLP-032	tagctgtcaagcgtggagcattcgcgccg	
NLP-033	tgctccacgcttgacagctagctcagtcctaggtataatactagtcgcc	
NLP-034	gtccatcatggtcaaaaaaagcaccgactcggtgccac	
NLP-035	ggtgctttttttgaccatgatggacgagctgaagtccatct	
NLP-036	cgcgaggcgtgcgaatcgatctccccgcacct	
NLP-037	atcgattcgcacgcctcgcgacctcgc	
NLP-038	gctgggcgcgcgacgcggtcctctacaaggagagaa	
NLP-039	ggagacgaccatatggacaagaagtactcgatcggcctgg	pLNS001
NLP-040	cccgttttcattcaatcgccgcccagctgg	

NLP-041	cggcgattgaatgaaaacgggcccccccct	
NLP-042	acttcttgtccatatggtcgtctccccaaaactcgagc	
NLP-043	cggcgagatgacctcatgctgttttagagctagaaatagcaagttaaaataaggc	pLNS005
NLP-044	gcatgaggtcatctcgccgactagtattatacctaggactgagctagc	
NLP-045	tatgacgtggtcggtccgg	pLNS002
NLP-046	gccatgatactgacctgcgaagcggctggacaggaac	
NLP-047	gttcctgtccagccgcttcgcaggtcagtatcatggccg	
NLP-048	ctgtcgggcgtctcattgc	
NLP-049	gcaatgagacgcccgacaggcgcggatccaaataaaacg	
NLP-050	ccggaccgaccacgtcataaaaaaaagcaccgactcggt	
NLP-051	gcacattatttcacttcgcagttttagagctagaaatagcaagttaaaataaggc	pLNS006
NLP-052	tgcgaagtgaaataatgtgcactagtattatacctaggactgagctagc	
NLP-053	tcgaagccgtacttggaaaggg	pLNS003
NLP-054	agaactccgtcggggtcgcggag	
NLP-055	ctccgcgaccccgacggagttctccgacgcgacgctgaag	
NLP-056	cgcttccgcaatcctgagatc	
NLP-057	atctcaggattgcggaagcggcgcggatccaaataaaacg	
NLP-058	cctttccaagtacggcttcgaaaaaaaagcaccgactcggtg	
NLP-065	ccgcgcgccgcctaagcagatctcaattggatatcggc	pLN080
NLP-066	ggcggcgcgcggcgcgcc	
NLP-067	agcgccaggaactggtcgaccgca	pLN081
NLP-068	tcctggcgctgatccagggcctgg	
NLP-069	cggtcggccaccttgggcgccgtcgagactgc	pLN082
NLP-070	gagatctgcttagctggaacgttcgtggcgttgg	
NLP-071	gaacgttccagctaagcagatctcaattggatatcggccggc	
NLP-072	cccaaggtggccgaccggtgcttg	
NLP-073	aggcaagggctaagcagatctcaattggatatcggccggc	pLN083
NLP-074	cagttcctgggtggccgaccggtgcttg	
NLP-075	gtcggccacccaggaactggtcgaccgcatccg	
NLP-076	agatctgcttagcccttgccttccgaggcg	
NLP-077	ccttctaagcagatctcaattggatatcggccg	pLN084
NLP-078	gcttagaaggccaaggccgagcgg	
NLP-079	agggctaagcagatctcaattggatatcggccg	pLN085
NLP-080	gcttagcccttgccttccgaggcg	
NLP-081	ccaccgtcgcgctgacgacgggc	pLN086
NLP-082	gcgacggtggccgaccggtgctt	
NLP-083	acgttccagcggcaaggccaagtcggcgaag	pLN088
NLP-084	gatctgcttaggcggcgcgcgg	
NLP-085	gcgcgccgcctaagcagatctcaattggatatcggccggc	
NLP-086	tggccttgccgctggaacgttcgtggcgttggttcaggcgc	
NLP-087	gtcggccaccatggctcagccaagtgtcgcg	pLN061
NLP-088	ccaagcttcttaggatggatgggagagagtcaacagaaaccag	
NLP-089	cccatccatcctaagaagcttggctgttttggcggat	

NLP-090	gctgagccatggtggccgaccggtgct	
NLP-091	gtcggccaccatgaacggattgcgatcgaaaacccaacag	pLN062
NLP-092	aattgagatctgcacactttctgtctgaaaagaccgggca	
NLP-093	gacagaaagtgtgcagatctcaattggatatcggccggc	
NLP-094	atccgttcatggtggccgaccggtgct	
NLP-095	gtcggccaccatggcgcagctcaacgtcgc	pLN063
NLP-096	ccaagcttcttagagcacttccaggaaaagtgtgtaacggttttcc	
NLP-097	ctggaagtgctctaagaagcttggctgttttggcggatgag	
NLP-098	gctgcgccatggtggccgaccggtgct	
NLP-099	gtcggccaccatgaacggatcgcgatccacttctcag	pLN064
NLP-100	aattgagatctgcacctcgatatgccagcggccc	
NLP-101	gcatatcgaggtgcagatctcaattggatatcggccggc	
NLP-102	atccgttcatggtggccgaccggtgct	
NLP-103	gtcggccaccatggccaacgaagcgcataaagaacc	pLN065
NLP-104	aagcttcttactagcgcgccatgcggg	
NLP-105	ggcgcgctagtaagaagcttggctgttttggcggatgagagaag	
NLP-106	cgttggccatggtggccgaccggtgct	
NLP-107	gtcggccaccatgagccagacggggccc	pLN066
NLP-108	tgagatctgctcagccccggcggtttgacg	
NLP-109	ccggggctgagcagatctcaattggatatcggccggccacgcg	
NLP-110	tctggctcatggtggccgaccggtgct	
NLP-111	gtcggccaccatggctgcgatcccaaaggtgc	pLN067
NLP-112	aagcttcttatcagcgcgggcgacgcg	
NLP-113	cccgcgctgataagaagcttggctgttttggcggatgag	
NLP-114	atcgcagccatggtggccgaccggtgct	
NLP-115	gtcggccaccatggagcggacggaacgcac	pLN068
NLP-116	attgagatctgctcacttcaccgccgccacgg	
NLP-117	cggtgaagtgagcagatctcaattggatatcggccggc	
NLP-118	tccgctccatggtggccgaccggtgct	
NLP-119	tcatcacatgtccgatcagtctcaagaacc	pLN087
NLP-120	cggacatgtgatgatgatgatgatggctgc	
NLP-121	acgttccagcgatgacgcgccggcggag	pLN089
NLP-122	ttggccttgccttcgtcgcgatcaaacaccggagc	
NLP-123	atcgcgacgaaggcaaggccaagtcggcgaagaaggaaaag	
NLP-124	gcgcgtcatcgctggaacgttcgtggcgttg	
NLP-125	gtcggccaccatgcgtaaaggcgaagagctgttcact	pLN090
NLP-126	gctaccactgccacctttgtacagttcatccataccatgcgtgatgcc	
NLP-127	gtacaaaggtggcagtggtagcatgtccgatcagtctcaagaacctacaatggagg	
NLP-128	ccgctctagattaggcgccgcgtcccc	
NLP-129	cggcgcctaatctagagcggccattcactggccgtcg	
NLP-130	ctttacgcatggtggccgaccggtgct	
NLP-131	gacgaagctgaataagccttaattaatatgcatggtaccttaagatctcgagctcc	pLN091
NLP-132	cttgctcaccatatggtcgtctccccaaaactcgagc	

NLP-133	gagacgaccatatggtgagcaagggcgaggagc	
NLP-134	gtcagtatcatggtggccgaccggtgct	
NLP-135	gtcggccaccatgatactgacctgcccggagtgc	
NLP-136	aattaaggcttattcagcttcgtccttgaaggcggt	
NLP-137	gagctgtacaagtaagccttaattaatatgcatggtaccttaagatctcgagctccg	pLN092
NLP-138	gctcgccttgacctgaatcgccagcggcatcagca	
NLP-139	cgattcaggtcaaggcgagcgtccgctagga	
NLP-140	cactgccaccggccagatcgccgctcgcc	
NLP-141	cgatctggccggtggcagtggtagcatggtgagc	
NLP-142	aattaaggcttacttgtacagctcgtccatgccgc	

SUPPLEMENTARY REFERENCES

8. Tan W, Cheng S, Li Y, Lu N, Sun J, Tang G, Yang Y, Cai K, li x, Ou X, Gao X, Zhao G-P, Childers W, Zhao W. 2022. Phase separation modulates the assembly and dynamics of a polarity related scaffold-signaling hub doi:10.21203/rs.3.rs-1398614/v1. Research Square.

9. Zhao W, Duvall SW, Kowallis KA, Tomares DT, Petitjean HN, Childers WS. 2018. A circuit of protein-protein regulatory interactions enables polarity establishment in a bacterium. bioRxiv doi:10.1101/503250:503250.

10. Ptacin JL, Gahlmann A, Bowman GR, Perez AM, von Diezmann AR, Eckart MR, Moerner WE, Shapiro L. 2014. Bacterial scaffold directs pole-specific centromere segregation. Proc Natl Acad Sci U S A 111:E2046-55.
